# NMD3 regulates both mRNA and rRNA nuclear export in African trypanosomes via an XPOI-linked pathway

**DOI:** 10.1093/nar/gkv330

**Published:** 2015-04-14

**Authors:** Melanie Bühlmann, Pegine Walrad, Eva Rico, Alasdair Ivens, Paul Capewell, Arunasalam Naguleswaran, Isabel Roditi, Keith R. Matthews

**Affiliations:** 1 Centre for Immunity, Infection and Evolution, Institute for Immunology and Infection Research, School of Biological Sciences, Kings Buildings, University of Edinburgh, West Mains Road, Edinburgh EH9 3JT, UK; 2 Centre for Immunology and Infection, Department of Biology, University of York, YO10 5DD, UK; 3 Institute of Cell Biology, University of Bern, CH-3012 Bern, Switzerland

## Abstract

Trypanosomes mostly regulate gene expression through post-transcriptional mechanisms, particularly mRNA stability. However, much mRNA degradation is cytoplasmic such that mRNA nuclear export must represent an important level of regulation. Ribosomal RNAs must also be exported from the nucleus and the trypanosome orthologue of NMD3 has been confirmed to be involved in rRNA processing and export, matching its function in other organisms. Surprisingly, we found that *Tb*NMD3 depletion also generates mRNA accumulation of procyclin-associated genes (PAGs), these being co-transcribed by RNA polymerase I with the *procyclin* surface antigen genes expressed on trypanosome insect forms. By whole transcriptome RNA-seq analysis of *Tb*NMD3-depleted cells we confirm the regulation of the *PAG* transcripts by *Tb*NMD3 and using reporter constructs reveal that *PAG1* regulation is mediated by its 5′UTR. Dissection of the mechanism of regulation demonstrates that it is not dependent upon translational inhibition mediated by *Tb*NMD3 depletion nor enhanced transcription. However, depletion of the nuclear export factors XPO1 or MEX67 recapitulates the effects of *Tb*NMD3 depletion on *PAG* mRNAs and mRNAs accumulated in the nucleus of *Tb*NMD3-depleted cells. These results invoke a novel RNA regulatory mechanism involving the NMD3-dependent nuclear export of mRNA cargos, suggesting a shared platform for mRNA and rRNA export.

## INTRODUCTION

The compartmentalization of nuclear DNA away from the translational apparatus in the cytoplasm of eukaryotic cells requires elaborate mechanisms to achieve the regulated export of RNA to enable gene expression (1,2). The multiple stages of nuclear export provide several potential regulatory steps to ensure that RNAs of different function can be held inactive until fully matured, localized or targeted for cytoplasmic degradation (3,4). This prevents incorrectly or incompletely processed RNAs from perturbing normal cellular functions by interfering with important cellular processes, or generating products that would be deleterious to the cell (5,6). Such RNAs can include nascent ribosomal RNA subunits, tRNAs, incorrectly processed mRNAs, nuclear RNAi targets that require trafficking to the cytoplasm for degradation, as well as bulk mRNAs. Hence, in addition to transcription, mRNA stability and translation, the export pathway for a variety of cellular RNAs provides considerable scope for the cell to control the expression and function of transcripts (4). Such regulatory pathways have been extensively studied in both yeast and mammalian systems and include complex rRNA maturation and export steps (7) and the nonsense-mediated decay (NMD) of incorrectly processed mRNAs (8).

African trypanosomes provide an interesting opportunity to dissect the pathways of gene regulation in evolutionarily divergent organisms (9,10). These organisms, important pathogens of humans and livestock in sub-Saharan Africa, diverged very early in the eukaryotic lineage and exhibit several novelties of gene expression that have attracted considerable attention. This includes their co-transcription of functionally unrelated genes as part of polycistronic transcription units (11) and their extreme reliance on post-transcriptional mechanisms of gene expression control necessitated by this genome organization (12). Particularly, mRNAs are generated from polycistronic transcription units by the coupled processes of 5′ *trans*-splicing of a 39-nt capped leader sequence and 3′ polyadenylation (13,14). These RNA processing steps allow trypanosomes to exploit the transcription of protein coding genes by RNA polymerase I in order to generate the high levels of surface protein transcripts necessary for their survival or fitness in the mammalian bloodstream or tsetse fly midgut (15,16). In the tsetse midgut, the surface coat is composed of different isoforms of procyclin. These isoforms (EP procyclin, GPEET procyclin) show differential expression during the parasite's maturation in the tsetse fly (17) and are transcribed from several transcription units on different chromosomes in the parasite genome (18). These are transcribed by RNA polymerase 1 (pol I) and contain unrelated genes of unknown function, namely the procyclin-associated genes (PAGs)(19,20). *Procyclin/PAG* genes are believed to be transcribed in the nucleolus since procyclic form parasites do not possess a detectable expression site body, an extranucleolar site of pol I transcription for the surface antigen genes of the bloodstream form (21). Although *PAG* transcripts are generated from the same transcription unit as the *procyclin* genes, their overall mRNA abundance is much lower (19,22–24).

The understanding of regulated gene expression in trypanosomes through the action of protein factors remains very incomplete. Nonetheless, the trypanosome genome encodes an abundance of RNA binding proteins, many of which are unique to these evolutionarily divergent organisms (12,25,26). The characterization of these kinetoplastid-specific factors has revealed a common role in mRNA stability and translation, often involved in regulating the development through the many stages that accompany progression of the parasites through their complex life cycle (27). In addition to these specific factors, trypanosomatids also encode a core set of conserved protein factors with predicted functions in gene expression. These include many well characterized components of the translational machinery, as well as proteins associated with RNA processing, degradation and nuclear export (28,29). In particular, bioinformatics analyses of the genomes of kinetoplastid parasites and other eukaryotic groups including a broad group of opisthokonta have revealed a general conservation of many components of the nuclear export machinery, which are responsible for the trafficking of cellular RNAs (rRNAs, mRNAs, tRNAs, miRNAs, snRNAs) to the cytoplasm (28). The Ran-GTP-dependent RNA export machinery is well conserved, with Ran orthologues in *Trypanosoma brucei* being over 70% identical to yeast and mammals and a divergent MEX67 orthologue with bulk mRNA export function has been recently characterized (29,30). Moreover, there exists particular conservation of the rRNA export machinery, with XPO1 and *Tb*NMD3 demonstrating over 50% similarity to their human counterparts. Other, more evolutionarily restricted components of this machinery, such as ArX1, Alb1p and PHAX, are not evident in the *T. brucei* genome but there are additional kinetoplastid-specific components that have been identified, most notably the developmentally regulated proteins P34/P37(31) and NOPP44/46(32). Hence, XPO1, *Tb*NMD3 and P34/P37 and NOPP44/46 cooperate to process and export rRNA, demonstrating the role of both highly conserved, and parasite-specific, components in this fundamentally important export pathway (33–37).

In this study, we have made the surprising discovery that post-transcriptional gene silencing by RNAi of trypanosome NMD3 results in a striking increase in *PAG* mRNAs in procyclic form trypanosomes, which is not mediated by the effects of NMD3 depletion on transcription, or translation. Rather, our results invoke a novel regulatory step dependent upon nuclear export of the mRNA, *PAG* transcripts representing an mRNA cargo with particular cytoplasmic instability whose trafficking is dependent on a nuclear export pathway conventionally involved in the maturation and export of large subunit ribosomal RNAs (38).

## MATERIALS AND METHODS

### Trypanosomes culture

For developmental expression analysis using pleomorphic trypanosomes, pleomorphic slender cells were harvested from a mouse 3 days post-infection, intermediate cells were harvested 4 days post-infection and stumpy cells were harvested 6 days post-infection. Bloodstream and procyclic parasites were grown in culture as described (39). For stable transfection 1 × 10^8^ procyclic form or 4 × 10^7^ bloodstream form cells were subjected to nucleofection with the Nucleofector system (Amaxa) using programs X-014 (PCF) or X-001 (BSF) as described in (40) and selected using the appropriate drugs: puromycin, 1–2 μg/ml (PCF) or 0.5 μg/ml (BSF), hygromycin: 20 μg/ml (PCF) or 2.5 μg/ml (BSF), phleomycin: 5–10 μg/ml (PCF) or 1 μg/ml (BSF).

RNAi lines of *TbNMD3* and *XPO1* were created using the stem loop vector pALC14 (41). Inserts were amplified by polymerase chain reaction (PCR) using primers detailed in Supplementary Table S1. For transfection pALC14 vectors were linearized with NotI prior to transfection.

### Report constructs

The constitutive CAT reporter construct was based on the expression vector pHD449 (42), as described in (43). The plasmid has a truncated *aldolase* 3′ untranslated region (UTR) providing constitutive expression in *T. brucei*.

### Chromatin immunoprecipitation

The chromatin immunoprecipitation (ChIP) protocol was adapted from (44). Depletion of *Tb*NMD3 was induced 3 days prior to the chromatin IP. RNAi cells were centrifuged at 2000 rpm for 10 min in a clinical centrifuge and washed once in phosphate buffered saline (PBS). The cells were resuspended in 900 μl PBS containing 1% paraformaldehyde and incubated at room temperature (RT) for 8 min. To stop the reaction 100 μl of 1.25 M glycine was added and cells were washed in 10 ml PBS after 5 min. The cells were lysed by resuspension in 0.25 ml of lysis buffer (50 mM Tris-HCl pH 8, 10 mM ethylenediaminetetraacetic acid (EDTA), 1% sodium dodecyl sulphate (SDS), Protease inhibitor cocktail (Sigma)) and sonicated 3 × 15 s on ice with 30 s recovery intervals on ice. The sample was transferred to a low binding eppendorf tube and rocked for 30 min at 4°C. After centrifugation at maximum speed for 10 min, the supernatant containing chromatin–DNA complexes was saved in PCR tubes.

For immunoprecipitation, 20 μl of dynabeads (Promega) were washed 3× in 200 μl RIPA buffer (10 mM Tris-HCl pH 7.5, 1 mM EDTA, 1% TritonX 100, 0.1% SDS 0.1% Na-deoxycholate, 140 mM NaCl). All steps were performed using low bind DNA eppendorf tubes. The dynabeads were resuspended in 200 μl RIPA buffer containing 2.4 μg of primary antibody (anti-histone H3 antibody, Abcam AB1791 or rabbit serum). Antibody-bead mixture was incubated for 2 h at 4°C rocking on rotor.

Chromatin-DNA was diluted 1:100 into RIPA buffer into a total volume of 200 μl, then added to the antibody-coated dynabeads and incubated overnight at 4°C with rocking. Then the beads were washed 1× in 200 μl RIPA buffer, 1× in high salt buffer (0.1% SDS, 1% Triton X100, 2 mM EDTA, 20 mM Tris-HCl pH 8, 500 mM NaCl) and 1× in LiCl buffer (0.25 M LiCl, 1% NP-40, 1% deoxycholic acid, 1 mM EDTA, 10 mM Tris-HCl pH 8), resuspended in 100 μl 10mM Tris 1mM EDTA pH 8.0 (TE) and transferred into a new eppendorf tube.

### ChIP DNA elution

For cross-link reversal, TE buffer was removed and the beads were resuspended in elution buffer (20 mM Tris-HCl pH 7.5, 5 mM EDTA, 50 mM NaCl, 0.1% SDS, 50 μg/ml proteinase K). The input chromatin-DNA was diluted 1:100 in elution buffer. These were left for 2 h at 68°C with rocking and vortexing at various intervals. Beads were captured and the supernatant was saved in a new tube. The DNA was cleaned up using a quick PCR clean up kit (Qiagen) and eluted with 50 μl dH_2_O. DNA concentration was determined using NanoDrop.

### Real time PCR

To analyse *PAG* locus transcription it was necessary to use primers for qRT PCR able to discriminate different regions of the *EP* and *GPEET* genomic loci. Although sequence similarity in the *procyclin* promoter region of the *EP* and *GPEET* gene loci prevented these regions being precisely differentiated by quantitative real time (qRT)-PCR assay, the limited up-regulation of each gene upon *Tb*NMD3 depletion meant that a specific up-regulation of *PAG* gene transcription would be discriminated, although the precise allele from which particular isoform transcripts were derived could not be accurately determined. Three dilutions (0.1, 0.01, 0.001 μg/reaction) of input DNA samples were used for standards. qPCR was performed with 0.001 μg DNA/reaction using MESA GREEN qPCR MasterMix Plus for SYBR® Assay (Eurogentec) in the ABI Prism 7000 Sequence Detection System (Applied Biosystems). The data were analysed using 7000 System SDS software v1.2 (Applied Biosystems). The obtained values were corrected with unspecific binding to rabbit serum and data were expressed as enrichment of DNA associated with immunoprecipitated H3 relative to a 1:100 dilution of input chromatin.

### Primer sequences

The following primer sequences were used for qRT-RCR analysis of Mex67 RNAi lines:

Mex67 Forw: ATCGCGTATACCGTGTGTCT; Mex 67 Rev: ACATGAAGAAATGGGCAGTG; SL sense: CGCTATTATTAGAACAGTTTCTGTAC; Alpha tubulin Rev: GTTACCAACCTGGCAACCA; RPL10 (Tb927.11.97.10) Rev: TGCAAATCAATGCTCTCCTT; RPL30 (Tb927.10.5120) Rev: GAGAACGTATTTGCCGGATT; TIM (Tb927.11.5520) Rev: CCGTTGCACTTCCAGTTG.

### RNA preparation and RNAseq analysis

RNA extraction and northern blot analysis were performed as previously described (43). RNASeq data (generated at BGI, Hong Kong) were analysed as follows. Firstly, raw fastq data were checked for quality using FastQC software (http://www.bioinformatics.babraham.ac.uk/projects/fastqc/). Next, the latest version of the *T. brucei* brucei 927 genome and annotation was obtained from: ftp://ftp.sanger.ac.uk/pub/project/pathogens/gff3/CURRENT/Tbruceibrucei927.gff3.gz on 19 February 2015. The gff format file was parsed to extract all gene transcripts coordinates; 8966 possessed 5′ UTR regions (total: 2 032 326 bases, average 227). Readsets were then grouped by sample type: 8 419 043 read pairs were obtained for PLUS and 19 340 407 read pairs for MINUS. Each sample readset (comprising several paired end sequence sets) was aligned independently to the *T. brucei brucei* 927 genome sequence using bowtie2 (v2.2.4) with parameters –very-sensitive -p 12 –trim5 13. The outputs were saved as sorted, indexed BAM files (using samtools v 0.2.0). Transcript coverage for each gene in each sample was then obtained using bedtools (v2.15.0) with the gene transcript coordinates extracted from the genome gff file. Counts were normalized to the aligned reads and re-expressed as log2 counts per transcript kilobase. Gene-level data were only retained if one or both of the sample tags exceeded the 10% quantile (45). Specific regions of interest (i.e. the *PAG* array) were selected and excised from the primary alignment files to calculate mapping statistics for that region only. RNAseq data sets have been deposited in the GEO database with accession number GSE67246.

### Preparation of riboprobes and drug treatments

In each case, genes for riboprobe preparation were inserted into pGEM-T easy vector containing T7/T3 or SP6 promoter sites flanking M13 forward and reverse primer binding sites as described previously (46). For drug treatment, Procyclic *Tb*NMD3 RNAi cells were induced with tetracycline for 3 days. A sufficient volume of 5 × 10^6^ cells/ml was cultured, so that for each time point there were 5 × 10^7^ cells available for RNA preparation and analysis. Two samples were prepared before any drug treatment, one of the induced and one of the uninduced cells. Induced and uninduced cells were treated the same way.

To inhibit transcription Actinomycin D (Sigma) dissolved in DMSO (dimethyl sulfoxide) was used at a final concentration of 5 μg/ml. To inhibit translation Cycloheximide (CHX) dissolved in dH_2_O was used at a final concentration of 50 μg/ml. To inhibit *trans-*splicing Sinefungin dissolved in dH_2_O was used at a final concentration 2 μg/ml. Leptomycin B (LMB) was supplied pre-dissolved in anhydrous ethanol and used at a final concentration of 0.1 or 1 μg/ml.

### CAT assay

The CAT ELISA assay (Roche) was performed according to manufacturer's instruction and as detailed in (43). CAT concentrations were determined from the standard curve giving the highest R-square value and the sample values being in the range of the standard.

### *In situ* hybridization

Induced and uninduced *Tb*NMD3 RNAi cells were harvested 62 h post induction and *in situ* hybridized as described (47) with the following modifications: after fixation, cells were incubated in 25 mM NH_4_Cl solution for 10 min and washed 2× with PBS. PBTSG (PBS + 2% triton, 0.5% saponin, 10 mg/ml glycine) was added to cells to permeabilize the parasite membrane and quench autofluorescence before cells were spread onto slides to settle in a humidity chamber for 20 min (at RT). Slides were washed 3× in PBS prior to prehybridization in Hybe B with/without unlabelled oligo dT. Hybridization was overnight as described with Cy3-labelled oligo dT[2 ng/ml]. Slides were washed once in 4XSSC;15% formamide, twice in 4xSSC, twice in 2XSSC, incubated in DAPI and mounted as described (47).

### Cell fractionation

Induced and uninduced *Tb*NMD3 RNAi cells were harvested 60 h post induction when cell counts were identical; aliquots for ‘Total’ cell were taken and cells were fractionated as described (47) with the following modifications: the cleared cell lysate was placed over 1 ml of 2 M sucrose and centrifuged at 10 000*g* (15 min at 4°C) to yield the S10 supernatant and P10 pellet (nuclei-enriched) fractions. This was repeated to clarify the fractions and buffer was added to equate the volume of the P10 fraction with that of the S10 fraction. Fractions were vortexed and 1/3 volumes were aliquoted for protein and 2/3 for RNA isolation. Subsequent cDNA production and qRT-PCR were as described (47). Western blots loaded 5 μl ‘Total’ protein and 10 μl ‘nuclear-enriched’ (P10) or ‘cytosolic’ (S10) fractions per lane. Anti-cPGK and anti-P0 were used at 1:200 and 1:5000 dilution, respectively, as described (48) and mouse anti-NUPI (a kind gift from Klaus Ersfeld; University of Bayreuth, Germany) was used at 1:1000 dilution on a 4–12% gradient gel (Invitrogen) with HRP-labelled anti-mouse at [1:25 000] (Sigma).

## RESULTS

**Figure 1.**
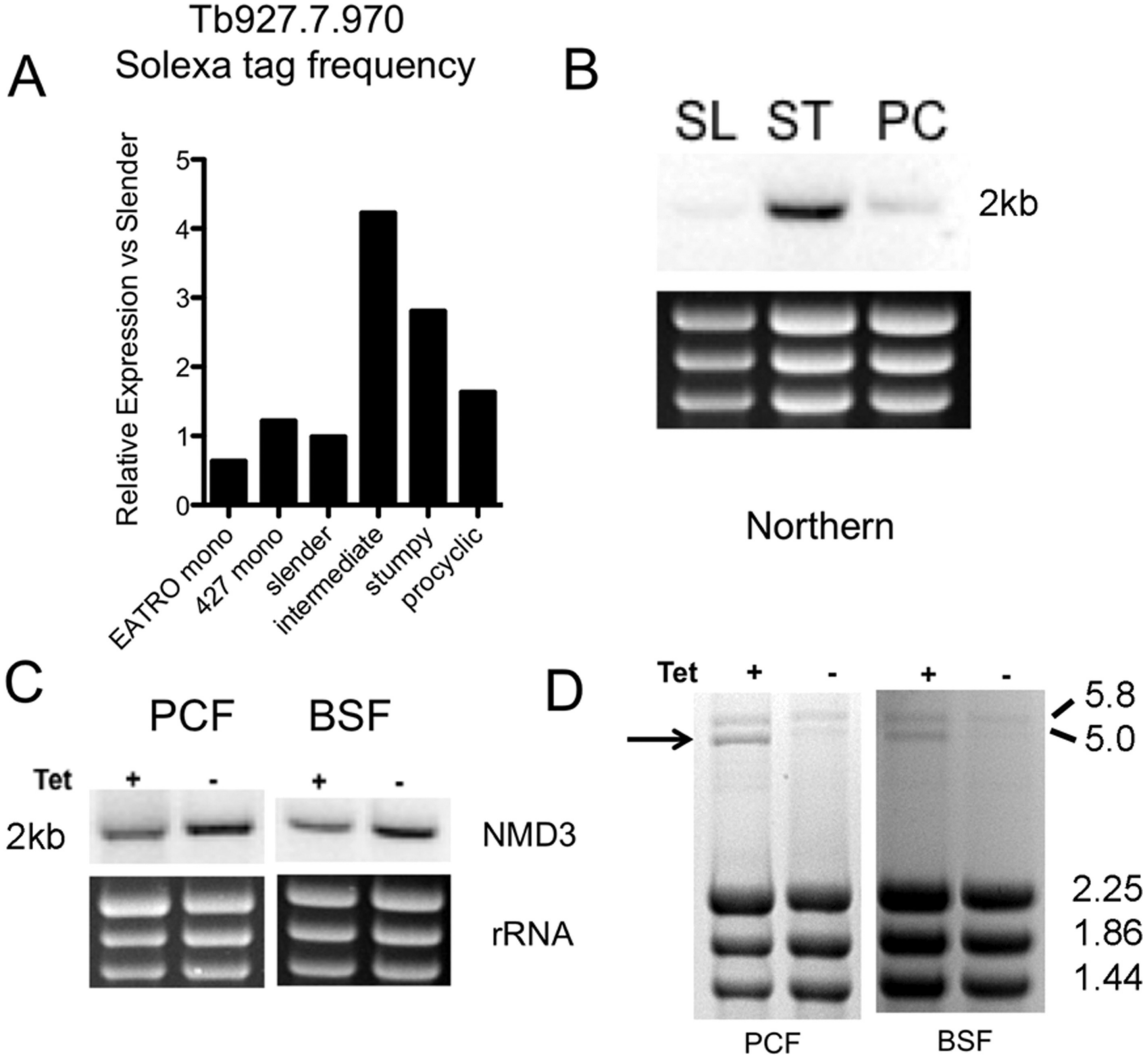
Expression of *Tb*NMD3 in different life cycle stages of *Trypanosoma brucei* and consequences of its knockdown by RNAi. (**A**) Relative transcript abundance of *Tb*NMD3 in two monomorphic bloodstream form strains (*T. brucei* EATRO 2340, *T. brucei* s427), pleomorphic strain slender, intermediate and stumpy forms (*T. brucei* AnTat1.1) and procyclic form trypanosomes (*T. brucei* 427), as determined by Digital SAGE analysis. Values are expressed relative to the expression level in pleomorphic slender cells (normalized to 1). (**B**) Northern blot of *Tb*NMD3 expression in *T. brucei* AnTat1.1 slender and stumpy forms and *T. brucei* 427 procyclic forms. (**C**) Northern blots of *TbNMD3* transcript levels in bloodstream and procyclic forms in the presence or absence of RNAi induction (the induced samples being derived 72 h after tetracycline addition). The *TbNMD3* transcript is depleted to 44% of its uninduced levels as assessed by RNA-Seq analysis of the procyclic form cell line. (**D**) Ethidium bromide stained RNA samples from bloodstream and procyclic forms induced, or not, to knockdown the expression of *Tb*NMD3. In each case, a 5-kb large subunit rRNA precursor accumulates upon *Tb*NMD3 depletion as expected for the functional depletion of this rRNA processing and export factor (34).

The *T. brucei* NMD3 homologue (Tb927.7.970) has been reported to be required for normal cell growth (34) and has been implicated in 60S rRNA processing and export (35), a role conserved in other eukaryotes. A role in conventional NMD is not expected for this molecule based on the precedent from other organisms (37). To investigate in more detail the biological role of NMD3 in *T. brucei* we initially analysed the expression profile of this gene in different life cycle stages. Specifically, mRNAs derived from two laboratory-adapted (monomorphic) slender cell populations (*T. brucei* s427, *T. brucei* EATRO 2340, these having lost the ability to generate stumpy transmission forms through serial passage) and from developmentally competent (pleomorphic) slender, intermediate and stumpy *T. brucei* AnTat1.1 cells were analysed by SOLEXA profiling (49), as was mRNA from s427 procyclic cells (Figure 1A). Interestingly this revealed that although the *TbNMD3* transcript abundance was similar in the monomorphic and pleomorphic slender cells, intermediate and stumpy forms demonstrated considerably enhanced levels (4.3- and 2.8-fold with respect to pleomorphic slender cells). Procyclic forms exhibited levels 1.6-fold those in pleomorphic slender forms (Figure 1A). These analyses were confirmed by northern blotting of total mRNA derived from pleomorphic slender and stumpy cells and from procyclic forms (Figure 1B).

To investigate the consequences of *Tb*NMD3 depletion for transcripts, RNAi lines were generated in both bloodstream and procyclic forms. In both cases the efficiency of knock down was incomplete (Figure 1C). Quantitation of procyclic forms by RNA-Seq (see later) demonstrated induced cells had retained 44% of the *Tb*NMD3 mRNA of uninduced cells. Nonetheless this was sufficient to generate the expected accumulation of a 5-kb large subunit rRNA precursor, this being readily detected in ethidium bromide stained total RNA from each life cycle stage (arrowed in Figure 1D). The accumulation of this rRNA processing intermediate upon *Tb*NMD3 depletion has been previously characterized (34). This was accompanied by an arrest in procyclic form parasite cell growth after 24–48 h, matching an earlier report ((34); Supplementary Figure S1), although cells remained motile and morphologically intact for at least 72 h. Bloodstream forms exhibited only a modest decrease in growth after *Tb*NMD3 RNAi was induced (Supplementary Figure S1), reflecting either less effective depletion in the derived cell lines or less dependence on *Tb*NMD3 levels at this life cycle stage. In consequence, all further studies were carried out in procyclic forms.

### Analysis of mRNAs regulated by TbNMD3 depletion

**Figure 2.**
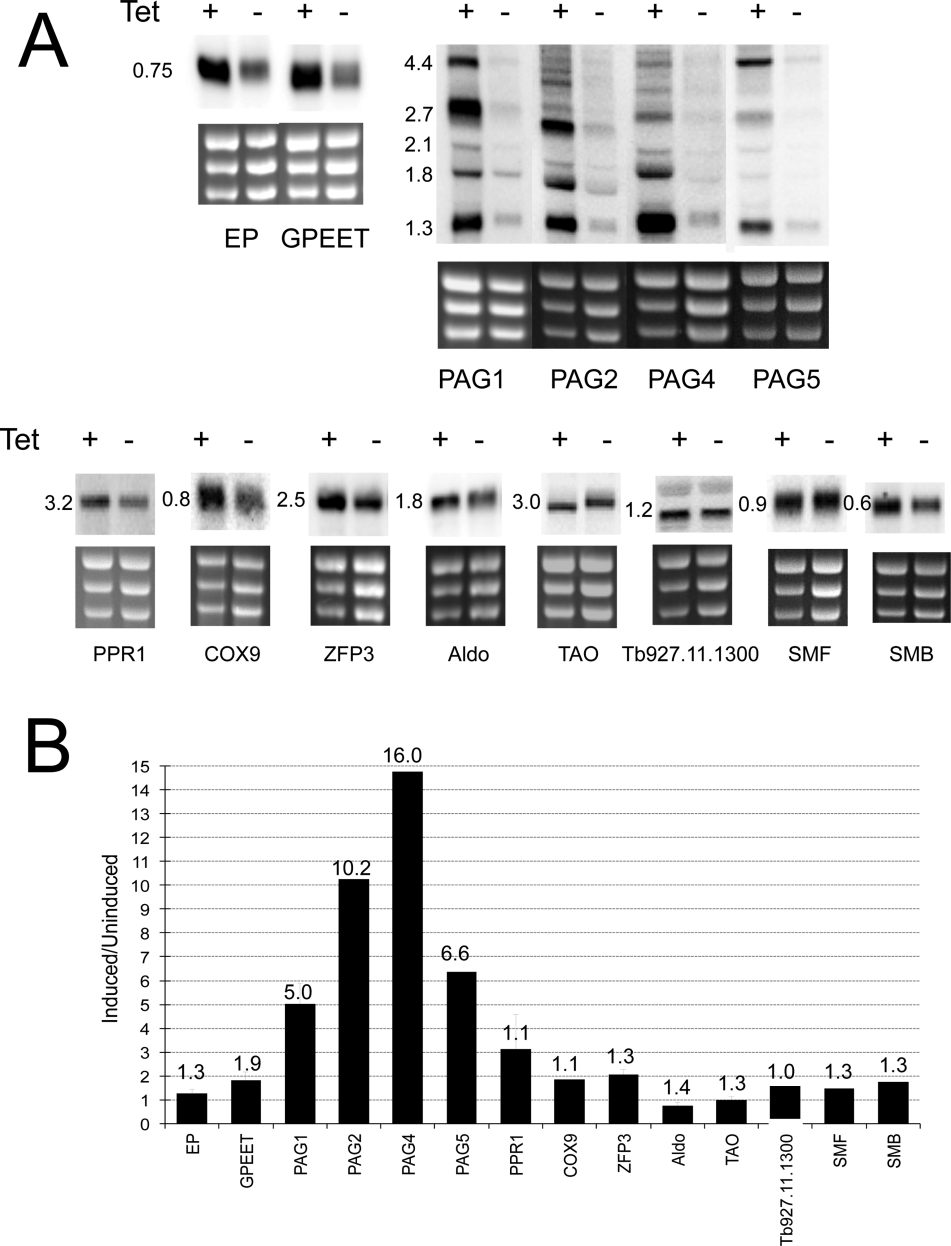
*Tb*NMD3 depletion in procyclic forms results in increased transcript levels for *PAG* transcripts. (**A**) Northern blots monitoring the expression of various transcripts in procyclic form cells depleted, or not, of *Tb*NMD3 by RNAi. In each case ethidium stained rRNA is shown below the northern image to indicate relative loading. For all samples, accumulation of the 5-kb rRNA precursors was observed in the induced sample, confirming effective *Tb*NMD3 depletion (not shown). (**B**) Abundance of several transcripts in procyclic forms depleted of *Tb*NMD3 quantitated from northern blots. In each case, the ratio of induced/uninduced samples is shown. *PAG* transcripts show particularly strong accumulation. The quantitation derived from subsequent, independent, RNA-Seq analysis is shown above the bar in each case as fold change with respect to the uninduced sample.

The enrichment of *TbNMD3* mRNA levels in stumpy forms prompted us to examine, in *Tb*NMD3-depleted procyclic forms, the mRNA levels for several genes regulated during the trypanosome developmental cycle or associated with differentiation events. Initially, we examined 14 RNA pol I and RNA pol II driven transcripts by northern blotting (Figure 2A). These included the genes for EP and GPEET *procyclin*, encoding the major surface protein expressed on procyclic forms and the genes for *PAG*1, *PAG*2, *PAG*4 and *PAG*5, which are co-transcribed with the *procyclin* genes in the same RNA pol I driven transcription units in the trypanosome genome (24) (Supplementary Figure S2). Additionally we examined genes for mitochondrial function (*PPR1*; *cytochrome oxidase subunit IX*; *Aldolase*; *Trypanosome Alternative Oxidase*, *TAO*), mRNA regulation and processing factors (*TbZFP3, SmF* and *SmB*) and a possible alternatively spliced mRNA, *Tb*927.11.1300 (45,50); S. Monk and K.R.M., unpublished observations). In each case riboprobes to the target gene were constructed and hybridized with total RNA from *Tb*NMD3 RNAi cells, induced or not, with detection of the rRNA precursor on ethidium stained gels confirming functional *Tb*NMD3 depletion in each case (data not shown).

Figure 2A demonstrates that there was a small increase in several of the analysed transcripts (1.1- to 1.9-fold through quantitative analysis; Figure 2B). However, most striking was the elevation of *PAG* gene transcripts (*PAG1, 2, 4, 5*), these being elevated 5–15-fold upon *Tb*NMD3 depletion (Figure 2A and B). The *PAG* transcripts are detected as a distribution of RNAs of various sizes whose characteristics and boundaries have been previously described in detail (22,24). In particular transcripts for PAG1 α (1231 nt excluding the SL and polyA tail) , β (1617 nt) and γ (2473 nt) are detected reflecting alternative splice sites in the PAG1 5′ UTR with an additional 4.4-kb band of unknown origin also being seen, as previously reported (20) (Supplementary Figure S2). Interestingly, although there was an increase in *PAG* transcripts in the induced samples, the profile of the bands detected did not substantially change; rather all detected species of mRNA were elevated upon *Tb*NMD3 depletion these increasing after 48 h (Supplementary Figure S3). One further notable change in the mRNAs analysed was detected, namely the size of the TAO transcript, which was slightly smaller in the *Tb*NMD3 RNAi induced sample (Figure 2A). Although we have not been able to establish the basis of this size change, it was very reproducible on distinct blots of individual RNA samples and, with the elevation of *PAG* transcripts and rRNA processing defect, was diagnostic of *Tb*NMD3 depletion.

**Figure 3.**
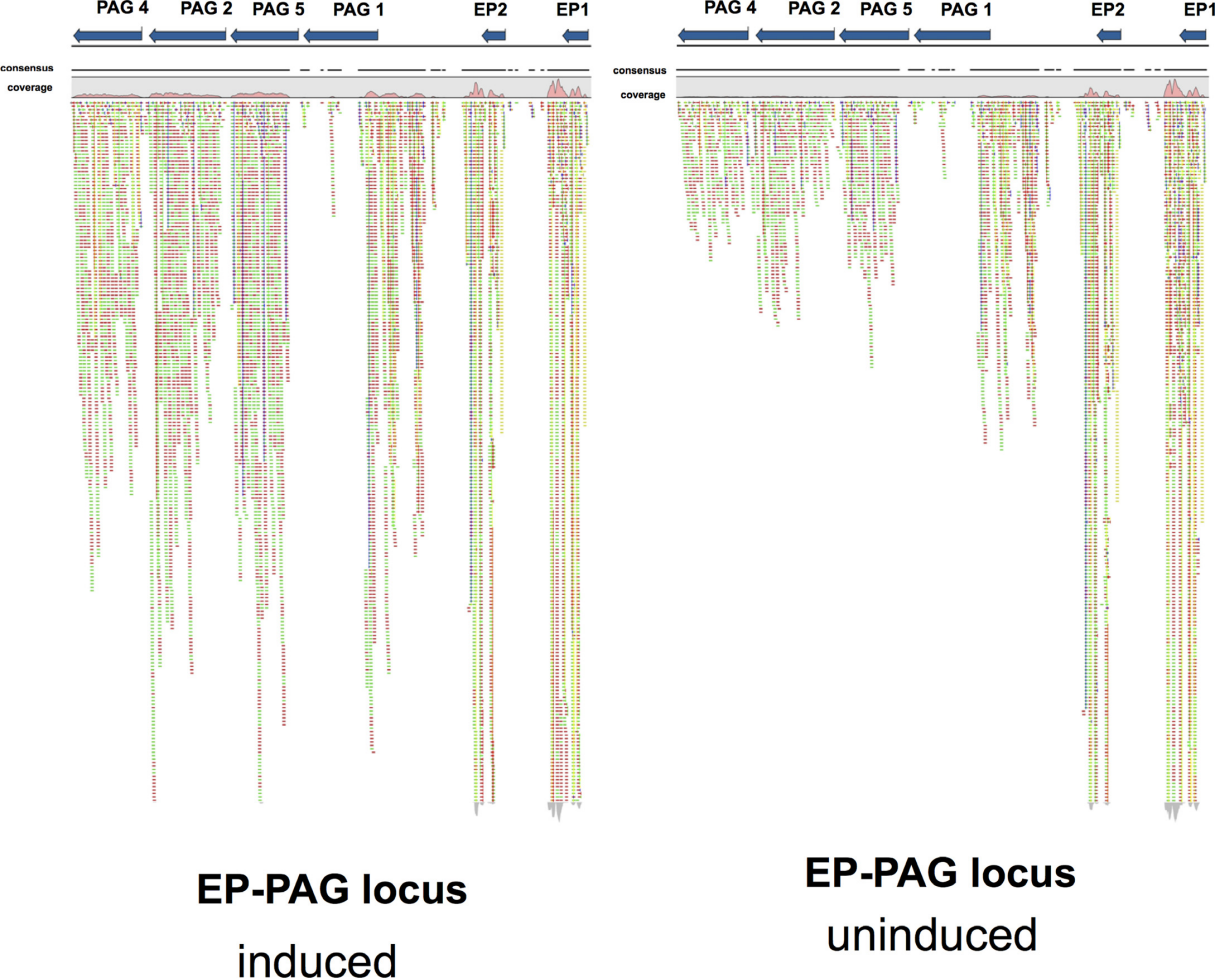
Regulation of transcripts derived from the *EP/PAG* expression locus. The genomic organization of the *PAG* and *procyclin* genes is shown at the top of the panel, with the alignment of RNA-Seq reads shown beneath. Although the *EP* transcripts do not change dramatically upon *Tb*NMD3 depletion, a strong upregulation of *PAG* transcripts is observed. The profile of the aligned reads however is unchanged.

To generate a higher resolution and genome-wide analysis of the effects of *Tb*NMD3 depletion in procyclic cells, polyA+ selected RNA from RNAi induced and uninduced samples was subject to RNA-Seq transcriptome sequencing. This confirmed the relative up-regulation of the *PAG* transcripts upon *Tb*NMD3 RNAi (Supplementary data 1). The *EP1-PAG* region (from the beginning of *EP1* to the end of *PAG*4; Figure 3) exhibited 2.45× more reads in the *Tb*NMD3 depleted cells compared to uninduced cells taking into account the total number of reads. The abundance but not the distribution of reads was changed indicating that particular processing events were not grossly affected. A further set of genes were also upregulated or downregulated (Supplementary data 1) although the *PAG* family transcripts were the most obviously represented, excluding VSG, ISG65, retroposon and expression site associated gene families. The quantitative correspondence with the northern blot analyses was good (Figure 2B), revealing the strong regulation of *PAG* transcripts, and more limited regulation of *GPEET* (1.9-fold elevated) and *EP* (1.3-fold elevated). Genes down-regulated comprised mainly hypothetical proteins and hypothetical conserved proteins (Supplementary data 1). From the small number of genes up-regulated 2-fold or more, there was a bias toward shorter transcripts perhaps related to similar observations with the depletion of *Tb*Puf2 and some other mRNA regulators (51).

### TbNMD3 depletion does not specifically enhance PAG gene transcription

The elevation of *PAG* transcripts upon *Tb*NMD3 depletion raised the possibility that *Tb*NMD3 might specifically repress the transcription of the *PAG* genes. In order to investigate this, a ChIP approach was pursued using an antibody against histone H3 to detect co-precipitated DNA as an indicator of transcriptional activity for specific gene loci (52). ChIP analysis of the *EP1/EP2* locus showed that there was reduced associated histone H3 upon *Tb*NMD3 depletion across the *PAG* locus but the same result was observed for the *procyclin* promoter region and also for a panel of other RNA pol II transcribed genes whose mRNAs were not elevated by *Tb*NMD3 depletion (Supplementary Figure S4A). Since histone H3 levels were unchanged in the respective cell lines (Supplementary Figure S4B) this indicated that *Tb*NMD3 depletion could generate a slightly decreased histone H3 association at multiple loci, but that this was not specific for the *PAG* transcripts and so could not account for their relative elevation.

### PAG regulation is mediated through the conserved 5′ untranslated region

**Figure 4.**
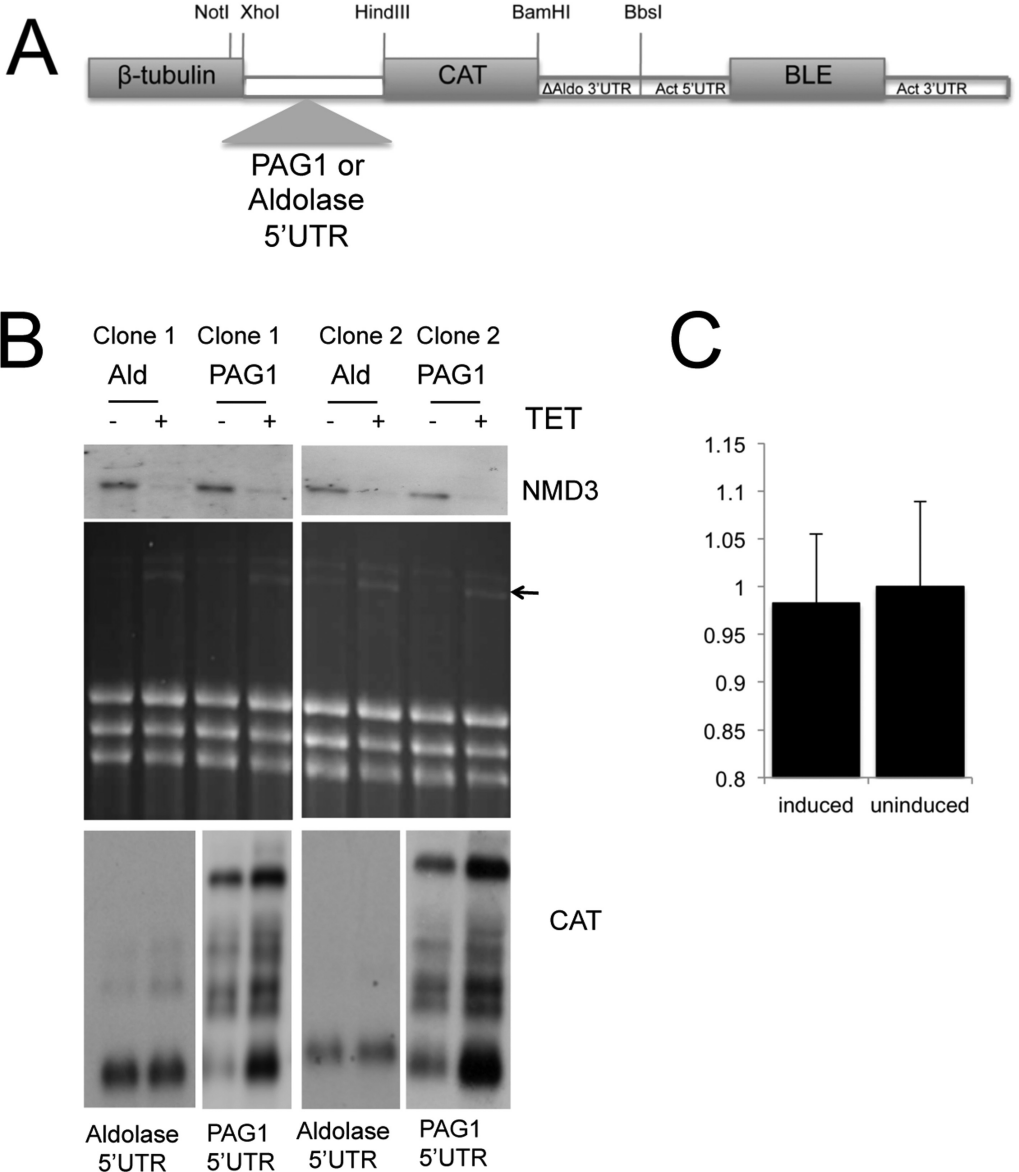
Reporter constructs with a *PAG* 5′ UTR are upregulated upon *Tb*NMD3 depletion. (**A**) Schematic diagram of the construct used to analyse the role of the *PAG*1 5′ UTR in *Tb*NMD3 depleted cells. As a control, the *aldolase* 5′ UTR was included as an alternative to the *PAG* 5′ UTR. (**B**) Northern blots of independent *Tb*NMD3 RNAi cell lines transfected with the CAT reporter comprising a *PAG1* 5′ UTR (Clone 1 PAG 5′ UTR, Clone 2 PAG 5′ UTR) or an *aldolase* 5′ UTR (Clone 1, Ald, Clone 2 Ald). RNAs from each line either induced or not to deplete *Tb*NMD3 are shown. Hybridization with probes detecting *TbNMD3* or *CAT* mRNA is shown, as is the ethidium bromide stained gel (revealing the rRNA processing intermediate, arrowed). (**C**) CAT protein levels from a *CAT-PAG1* 5′ UTR reporter cell line in which cells were either induced, or not, to deplete *Tb*NMD3. No difference in protein expression was observed despite increased *PAG* mRNA upon NMD3 RNAi induction.

The observation that *PAG* transcripts were upregulated in response to *Tb*NMD3 RNAi prompted us to investigate whether *PAG* regulatory signals could be responsible. Although in trypanosomatids 3′ UTR sequences frequently regulate gene expression, polyadenylation occurs immediately after the stop codon of the *PAG* genes, with regulation dominated by 5′ UTR sequences (24). Hence, a reporter construct (Figure 4A) was generated in which the *chloramphenicol acetyl transferase* (*CAT*) gene was linked to the *PAG*1 5′ UTR and integrated into the RNA pol II-transcribed *tubulin* gene locus of the *Tb*NMD3 RNAi cell line. A construct with an aldolase 5′ UTR was also generated as a control. The level of *CAT* expression was then analysed when *Tb*NMD3 was depleted or not. Figure 4B demonstrates that, as with the endogenous *PAG1* gene, several transcript isoforms of the *CAT-PAG1* 5′ UTR reporter line were increased upon induction of *Tb*NMD3 RNAi. This effect matched the response of the endogenous *PAG* transcripts, whereas the control reporter line with the aldolase 5′ UTR showed a relatively small increase in *CAT-aldolase* mRNA. At the protein level, CAT-ELISA assays demonstrated that the levels of CAT protein remained unchanged in the *CAT-PAG1* reporter line (Figure 4C). Therefore, the *PAG* 1 5′ UTR is sufficient to confer *Tb*NMD3 responsiveness to an unrelated reporter gene in a transcription unit driven by RNA pol II rather than RNA pol I. However, this elevated transcript abundance does not generate an equivalent CAT protein increase.

### PAG transcript regulation in response to inhibitors of distinct steps in gene expression

**Figure 5.**
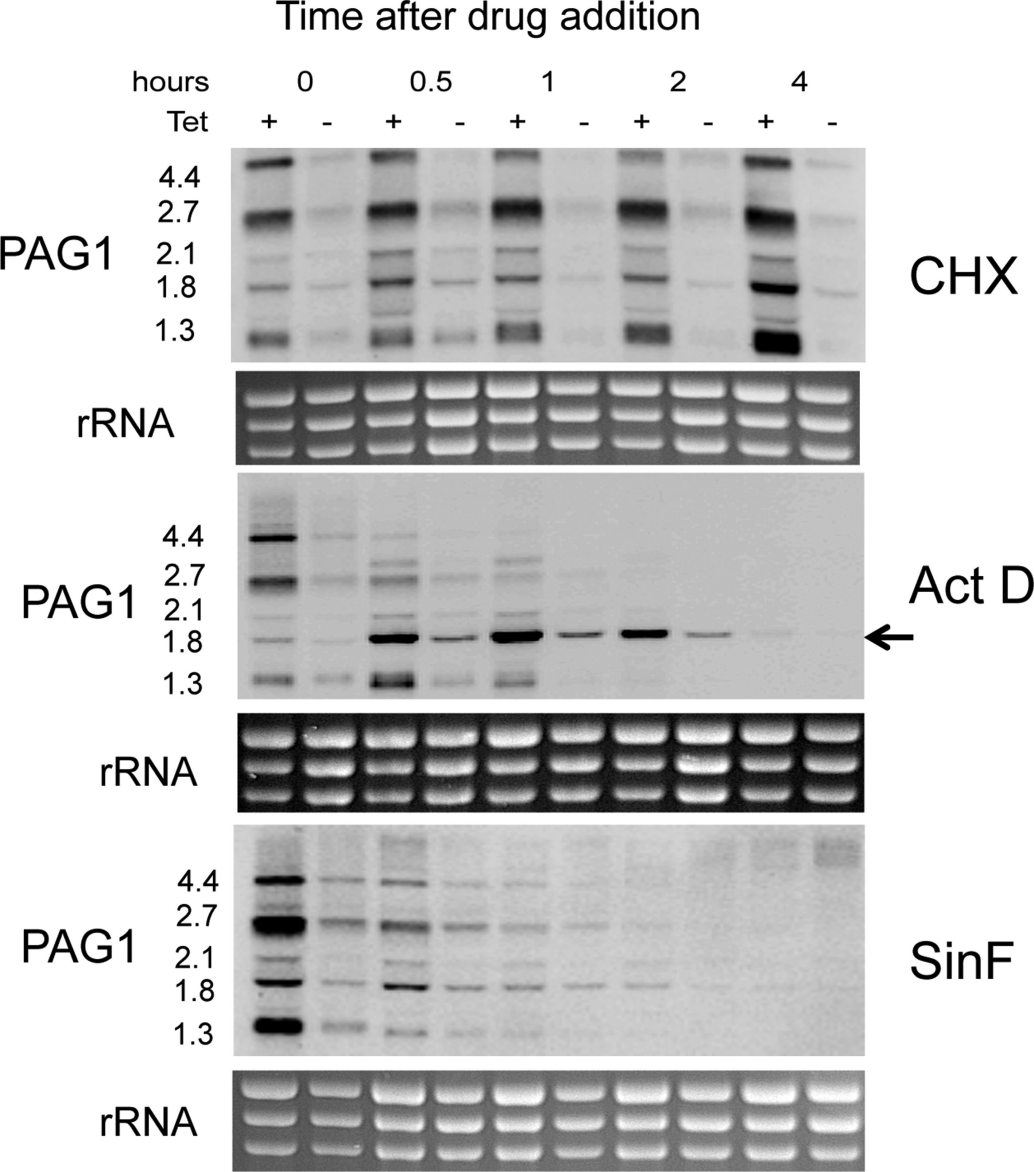
Effect of various inhibitors of different steps in the gene expression pathway on cells induced, or not, to deplete *Tb*NMD3. Cells in which *Tb*NMD3 was depleted, or not, were incubated in the presence of Cycloheximide, Actinomycin D or Sinefungin. RNA samples were isolated at various time points after drug addition and then northern blots assayed for *PAG1* transcript levels. In each case, the equivalent ethidium bromide stained rRNA bands are shown to indicate relative loading and RNA integrity.

We next investigated the basis of *PAG* transcript regulation following *Tb*NMD3 depletion by analysing different control steps in the gene expression pathway. Specifically we sought to block mRNA translation, transcription and RNA processing using Cycloheximide (CHX), Actinomycin D (ActD) and Sinefungin (SinF), respectively, in order to determine if any of these inhibitors would recapitulate or reverse the effect of *Tb*NMD3 depletion on *PAG* transcript levels (Figure 5 and Supplementary Figures S5 and S6). Given the effect of *Tb*NMD3 depletion on 60S rRNA processing and hence, potentially, translation we initially analysed the effect of Cycloheximide (Figure 5, ‘CHX’). However, this generated no dramatic change in the profile or abundance of the *PAG* transcripts in *Tb*NMD RNAi cells, although the abundance of *PAG* mRNAs increased in the induced samples somewhat over time (the effect on other transcripts is shown in Supplementary Figure S5). Using Actinomycin D (Figure 5, ‘Act D’), in contrast, resulted in a loss of longer *PAG* transcripts and accumulation of a smaller major transcript at 1.8 kb (arrowed on Figure 5, ‘ActD’), which then remained stable in both *Tb*NMD3 RNAi induced and uninduced samples. This suggested that inhibiting transcription caused longer *PAG* transcripts to be processed to a shorter stable form or that longer isoforms were preferentially degraded, but this occurred irrespective of *Tb*NMD3 levels. Finally, we analysed the effect of inhibiting *trans*-splicing using Sinefungin (Figure 5, ‘SinF’). Whilst no change in the profile of *PAG1* RNA was detected in the induced or uninduced samples, the elevated abundance of the *PAG1* transcripts in the *Tb*NMD3 depleted cells was quickly reversed; the *PAG* mRNA profiles of the uninduced and induced samples were equivalent within 1 h. Hence, inhibiting *trans*-splicing counteracts the enhanced levels of *PAG* transcripts generated by *Tb*NMD3 depletion.

### Inhibiting XPO1 recapitulates the NMD3 RNAi phenotype

Since inhibiting various steps in the gene expression pathway had not recapitulated the NMD3 effect on the *PAG* transcripts, we also analysed the export of nuclear RNAs. In eukaryotes, NMD3 and the nuclear export factor XPO1 interact, with NMD3 providing a chaperone to move rRNA cargos to the cytoplasm. In trypanosomes, XPO1 action has been analysed by drug inhibition using LMB (53,54) and by RNAi, revealing a role in nuclear export of the 7SL RNA, a component of the signal recognition particle (55). Therefore, we initially analysed the effect of the XPO1 inhibitor LMB at either 1 μg/ml or 0.1 μg/ml, these concentrations following earlier publications using this drug (53,55). Remarkably when 1 μg/ml of LMB was used (Supplementary Figure S7), but not 0.1-μg/ml LMB (data not shown), a recapitulation of the *Tb*NMD3 RNAi phenotype was observed in procyclic forms. Thus, *EP* and *GPEET* mRNAs were slightly elevated, whereas *PAG* mRNAs were more strongly upregulated (Supplementary Figure S7A). Furthermore, analysis of the *TAO* transcript revealed that the subtle size change in that transcript was also observed with LMB treatment (Supplementary Figure S7B).

**Figure 6.**
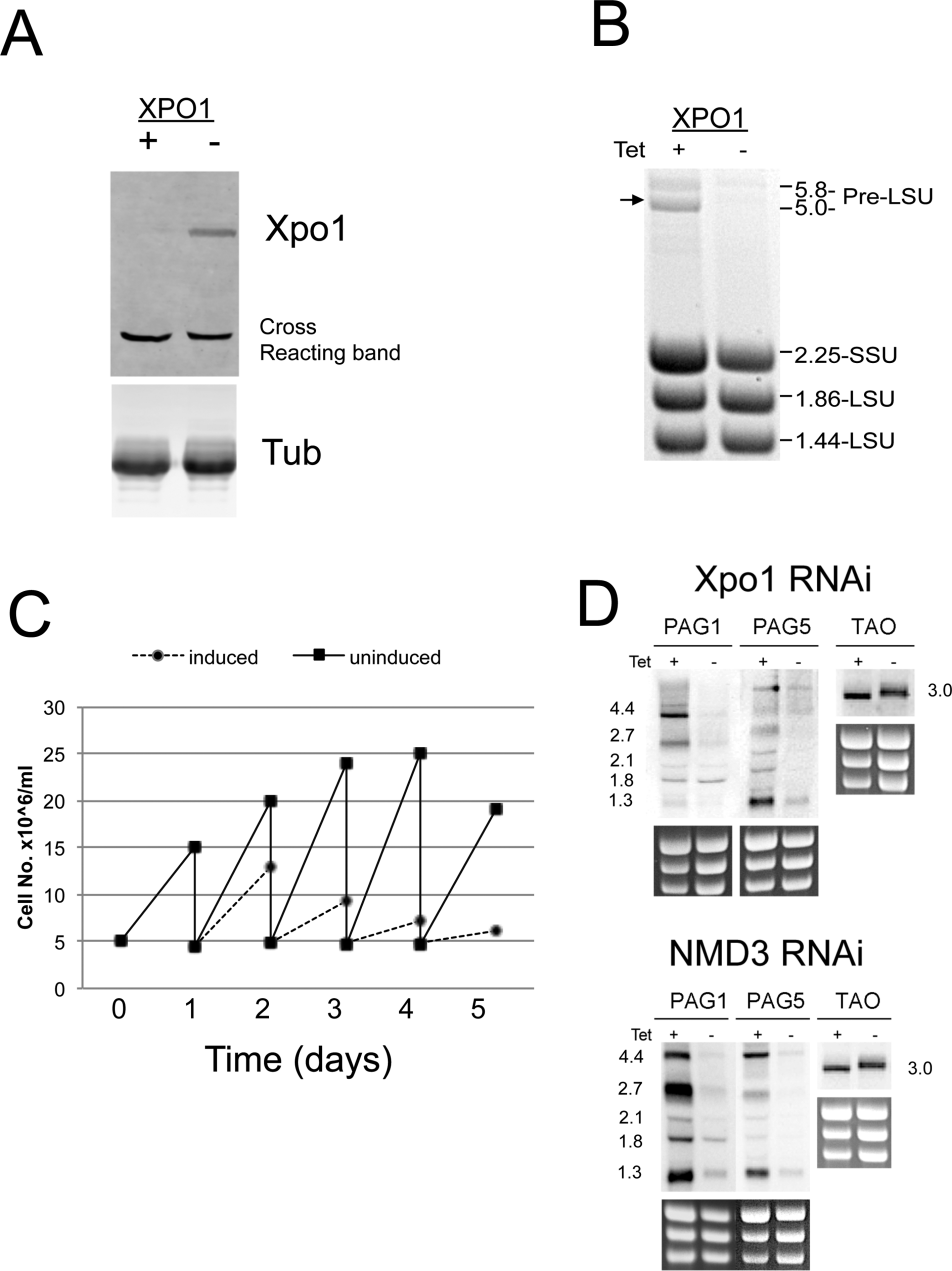
RNAi depletion of XPO1 perturbs rRNA processing. (**A**) Western blot of XPO1 levels in cells induced to undergo XPO1 RNAi, or not. A cross reacting band detected with the XPO1 detecting antibody reveals equivalent loading in each lane. (**B**) Ethidium bromide stained total RNA derived from the XPO1 RNAi line, demonstrating that the induced cells accumulate the 5-kb rRNA precursor, indicating perturbed rRNA processing and nuclear export similar to that observed under *Tb*NMD3 depletion. (**C**) Growth profiles of XPO1 RNAi cells induced or not with tetracycline. RNAi induction results in reduced growth after 24–48 h. (**D**) *PAG* transcripts are upregulated in the XPO1 RNAi line induced with tetracycline. Northern blots are shown detecting the abundance of transcripts when XPO1 (upper panel) or *Tb*NMD3 (lower panel) is depleted. Upon XPO1 or *Tb*NMD3 depletion, the changes in *PAG* transcript abundance and *TAO* transcript size characteristic of NMD3 depletion were also observed. In each case ethidium bromide stained rRNA indicates the relative loading.

Given that LMB may lack specificity in *T. brucei* or inhibit multiple targets, we sought to validate the results by depleting XPO1 in procyclic form trypanosomes by RNAi. Therefore, an *XPO1* gene fragment was inserted in to the pALC14 stem loop vector and the stable cell lines selected. Analysis of XPO1 using an antibody to this protein (kindly provided by Noreen Williams, Buffalo, NY, USA) confirmed its depletion upon the induction of RNAi (Figure 6A). Functional depletion was supported by the accumulation of the 5-kb precursor of the LSU rRNA in total RNA (Figure 6B) and the observation that the induced cells showed a strong growth phenotype over 48 h (Figure 6C).

Having validated the *XPO1* RNAi line, the consequences for the regulation of transcripts perturbed by *Tb*NMD3 depletion were assayed. Northern blots detecting *PAG*1 and *PAG*5 and *TAO* RNAs were analysed in parallel from the derived *Tb*XPO1 and *Tb*NMD3 RNAi lines (Figure 6D), demonstrating that the depletion of either target generated equivalent responses for each test transcript. Thus *PAG*1 and *PAG*5 were both elevated in the XPO1 line, albeit to a lesser extent than in *Tb*NMD3 depleted cells, and *TAO* demonstrated the characteristic size shift previously observed with *Tb*NMD3 depletion. This suggested that the *Tb*XPO1 and *Tb*NMD3 RNAi both generate equivalent mRNA changes, implicating nuclear export as the important point of control.

### mRNA transcripts are enriched in the nucleus upon NMD3 depletion

**Figure 7.**
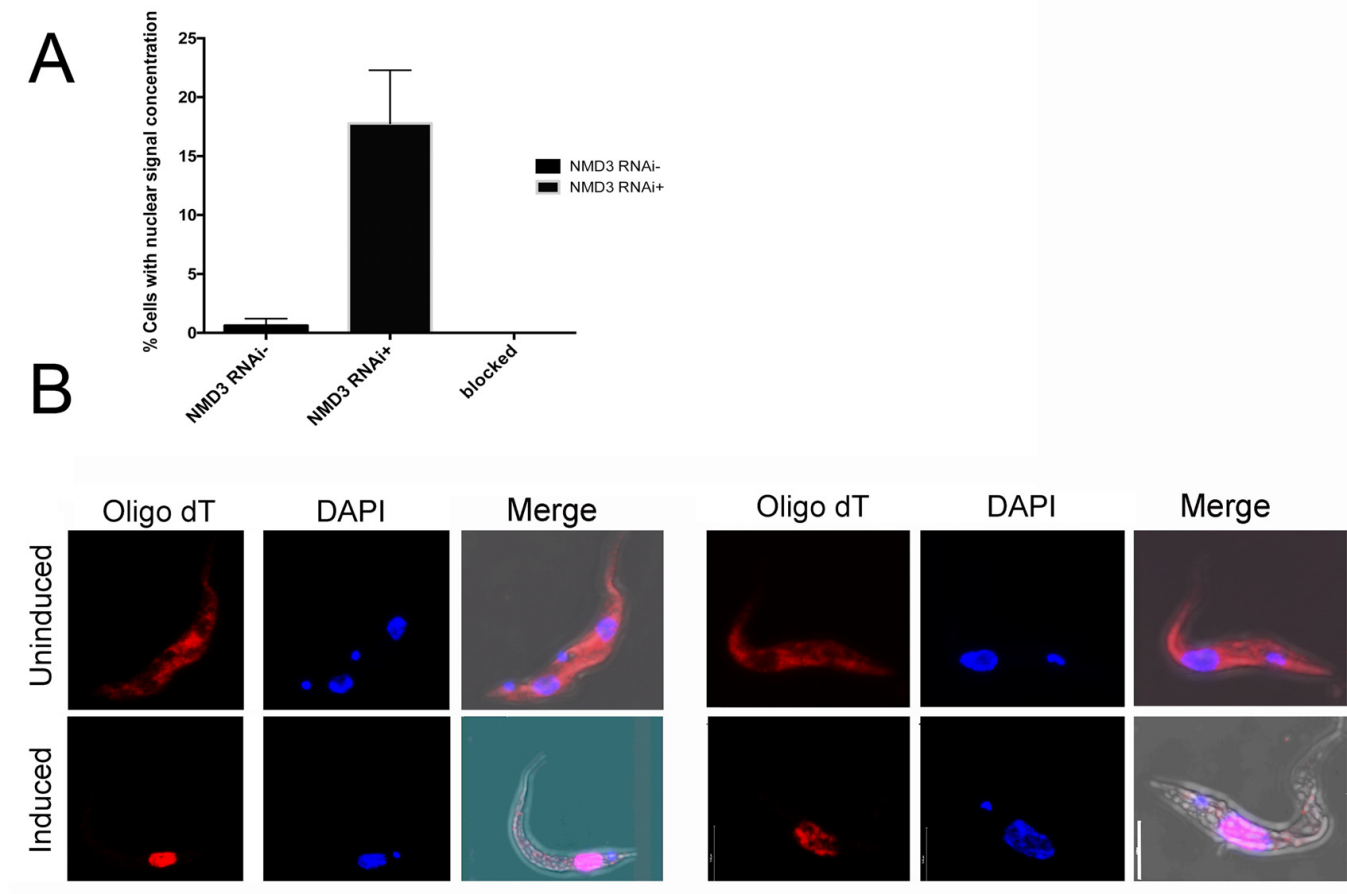
*In situ* hybridization of Cy3 labelled Oligo (dT) on cells induced, or not, to deplete *Tb*NMD3 by RNAi. (**A**) Quantitation of cells exhibiting a nuclear accumulation of Cy3 Oligo (dT) in *Tb*NMD3 RNAi cells or cells where hybridization is blocked with unlabelled Oligo (dT). (**B**) High magnification images of cells from the induced or uninduced population demonstrating either a nuclear accumulation or cytoplasmic distribution of the Cy3 signal. DAPI shows the position of the cell nucleus and kinetoplast whilst merged images show the combined Cy3, DAPI and phase contrast image. Pearson's correlation was significant for the colocalization of RNA and DNA in cells induced (*r* = 0.687) versus uninduced (*r* = 0.160) for *Tb*NMD3 RNAi. This significance was reduced (*r* = 0.431 versus *r* = 0.150, respectively) in large field images of ≥20 trypanosomes due to varying stages of *Tb*NMD3 protein decay in the population (Supplementary Figure S8). Bar=10 µM.

**Figure 8.**
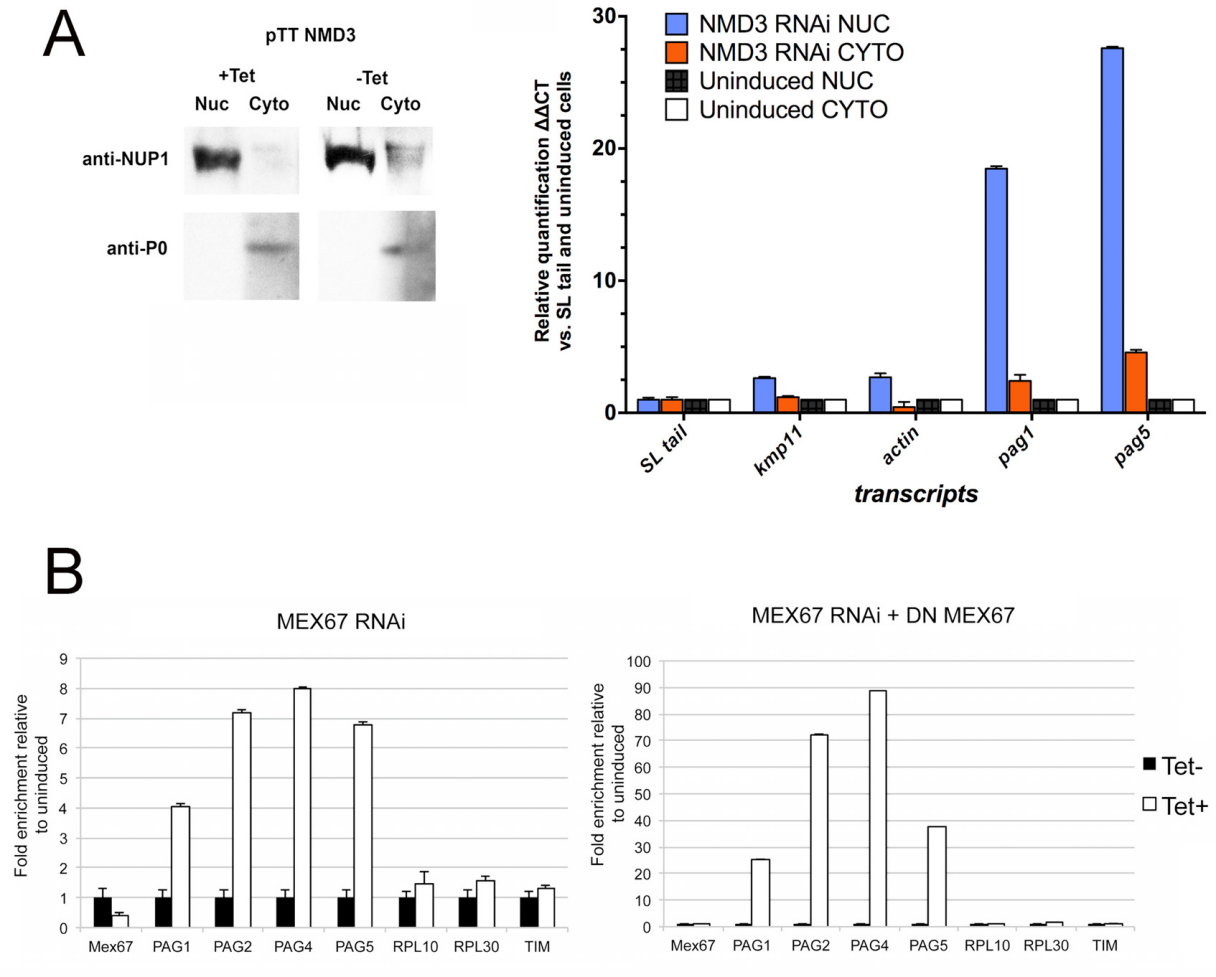
Nuclear accumulation of transcripts upon *Tb*NMD3 depletion. (**A**) Cell fractionation of *Tb*NMD3 RNAi cells 60 h after induction. Nuclear and cytoplasmic fractions were probed with the nuclear marker NUP1 and the cytoplasmic marker P0 to confirm effective fractionation. qRT-PCR was performed on mRNAs from the respective fractions, detecting the distribution of several mRNAs with nuclear accumulation being seen. (**B**) Relative levels of *MEX67*, *PAG1, 2,4, 5, RPL10, RPL30* and *TIM* mRNAs in cells where MEX67 is depleted by RNAi or where the RNAi is combined with expression of a dominant negative (DN) form of MEX67 lacking its N terminus. Alpha tubulin was used for normalization.

To investigate export of mRNAs from the nucleus we initially analysed the distribution of polyA+ RNAs in *Tb*NMD3 depleted cells by *in situ* hybridization. We predicted that if *Tb*NMD3 was involved in mRNA export from the nucleus we would observe a concentrated signal in the nucleus, although stable cytoplasmic mRNA would also be detected, depending on the kinetics of *Tb*NMD3 depletion and cytoplasmic mRNA turnover. To assist visualization of any changed distribution, cells were induced for *Tb*NMD3 depletion by RNAi over 62 h, once growth was reduced but before significant cell death was observed in the population (Supplementary Figure S1). Thereafter, the distribution of polyA+ RNA was compared in the induced and uninduced populations using Cy3-OligodT, with an unlabelled OligoT blocking step providing a control for specificity of the signal. Figure 7 shows that the induced population showed ∼20% cells with a strong nuclear concentration of Cy3 OligodT signal 62 h after induction, contrasting with less than 1% in uninduced cells, where the distribution of signal was non-nuclear. Cells pre-blocked with unlabelled OligodT showed little or no signal, confirming probe specificity (Supplementary Figure S8). Since this global polyA+ mRNA analysis suggested that all mRNAs might be accumulated in the nucleus, rather than *PAG* transcripts specifically, we analysed the distribution of several mRNAs after cell fractionation (Figure 8A). Specifically, nuclear and cytoplasmic fractions of parasites where *Tb*NMD3 was depleted or not were analysed for the relative distribution of *PAG1, PAG5, KMP11* and *actin*, with normalization to the 140-nt tail of the spliced leader mRNA. This revealed relative enrichment of all mRNAs in the nuclear fraction relative to SL RNA after *Tb*NMD3 depletion, with *PAG1* and *PAG5* particularly abundant, either through preferential retention or cytoplasmic instability. To provide further insight into this, we analysed the abundance of *PAG* mRNAs in a *MEX67* RNAi line, and in a cell line where a dominant negative (DN) N-terminal truncation of MEX67 is overexpressed in an RNAi background, this providing a stronger depletion effect (these cell lines being kindly provided by Bernd Schimanski, Bern, Switzerland) (29). Under these conditions bulk mRNA export from the nucleus is blocked (29). Figure 8B demonstrated that upon *MEX67* RNAi, there was a strong (up to 8-fold) increase in abundance of the *PAG* transcripts similar to that seen upon NMD3 depletion, in contrast to the abundance of the control transcripts *TIM, RPL30* and *RPL10*. Moreover this effect was strongly enhanced by combined RNAi and the expression of the dominant negative MEX67 protein (Figure 8B, right-hand panel).

Combined these results indicate NMD3 depletion causes an accumulation of mRNA in the nucleus, a consequence being increased abundance of *PAG* transcripts that are normally quickly degraded in the cytoplasm.

## DISCUSSION

In the experiments reported here we have demonstrated that depletion of the *T. brucei* orthologue of NMD3, functionally verified through *rRNA* processing defects, results in a significant up-regulation in *PAG* mRNAs (28-32). This regulation generated ∼5- to 16-fold increases, whereas only a few other cellular mRNAs were strongly elevated or decreased under the same conditions. The *PAG* genes are located downstream of, and cotranscribed with, the *EP* and *GPEET* genes by RNA Pol I (19). Despite this close linkage, *EP* and *GPEET* transcripts were only modestly up-regulated upon NMD3 depletion, perhaps because these transcripts are already ∼100-fold elevated over *PAG* transcripts.

The regulation of *PAG* transcripts and the effect of *PAG* sequences on adjacent gene transcripts are complex. *PAG* genes lack any significant 3′ UTR sequences with polyadenylation of *PAG1* occurring within 10 bases of the stop codon. Thus, regulation is believed to operate through their extensive 5′ UTR sequences and coding regions, which contribute to the low abundance of the *PAG* transcripts despite their transcription by RNA pol I (19,22,24). Accordingly, the *CAT*-*PAG*1 5′ UTR reporter construct was up-regulated upon *Tb*NMD3 RNAi, analogous to the regulation of the endogenous *PAG* transcripts, whereas a control mRNA was not significantly up-regulated. Hence, the observed regulation was specific to the *PAG* regulatory sequence but not dependent on the genomic location or the polymerase driving their transcription.

In order to investigate the basis of the upregulation of *PAG* transcripts in *Tb*NMD3 depleted cells, we analysed several steps in the gene expression pathway. Since analysis of the *PAG* 5′ UTR has demonstrated that it can act as a transcriptional silencer for nearby genes (22) we examined the interaction between *Tb*NMD3 depletion and transcription, specifically the histone occupancy of the *PAG* genes and several additional genes. Although a slight general decrease in histone occupancy was observed, this was not specific for *PAG* genes and was observed across a wide range of genomic loci. Hence we do not consider that transcription is a significant contributor to the effects observed. Thereafter we examined the contribution of several post-transcriptional steps to the regulation of *PAG* transcripts in *Tb*NMD3-depleted cells. We investigated whether the role of *Tb*NMD3 in rRNA maturation and translation could have resulted in a feedback upregulation of *PAG* mRNA, perhaps as cells attempted to compensate for reduced *Tb*NMD3 protein levels. However, CHX treatment did not generate an equivalent effect as *Tb*NMD3 depletion in uninduced cells. Similarly, when transcription was inhibited with Actinomycin D, the different length forms characteristic of the *PAG*1 transcripts were reduced to a major short transcript; however this was unrelated to *Tb*NMD3 depletion. Finally, to investigate the effect on mRNA processing via *trans*-splicing, we treated cells with Sinefungin. Interestingly, the up-regulation of *PAG* transcripts seen upon *Tb*NMD3 RNAi was reversed within 1 h, suggesting that this drug can counteract the depletion of *Tb*NMD3, destabilizing the elevated *PAG* transcripts or preventing their accumulation.

Having ruled out a direct role for transcription, RNA processing or translation as being responsible for *PAG* gene upregulation, we next turned our attention to nuclear export. Given that NMD3 cooperates with XPO1/CRM1 to export rRNA (35,56), we first investigated the effects of LMB on cells (an inhibitor of XPO1/CRM1 (57)) and then XPO1 RNAi. This revealed that the phenotypes resulting from *Tb*NMD3 depletion were recapitulated, supporting functional nuclear export as the potential mechanism for *PAG* mRNA regulation. An involvement of XPO1/NMD3 in *PAG* regulation was a surprise since these molecules are known to cooperate in the export of rRNA but not mRNA. One explanation was that the *PAG* transcripts might be exported for degradation via a discrete pathway. The positioning of the *PAG* genes is often close to the boundary of divergent or convergent transcription units or where there is a switch from RNA pol I to pol II mediated transcription (22). Although such regions have the potential to generate transcriptional overlap and resultant dsRNA, such that they might be templates for cytoplasmic RNAi mediated through the action of *Tb*AGO1 (58), we consider this unlikely since *PAG* levels are not elevated in *Tb*AGO1 deficient cells (22).

**Figure 9.**
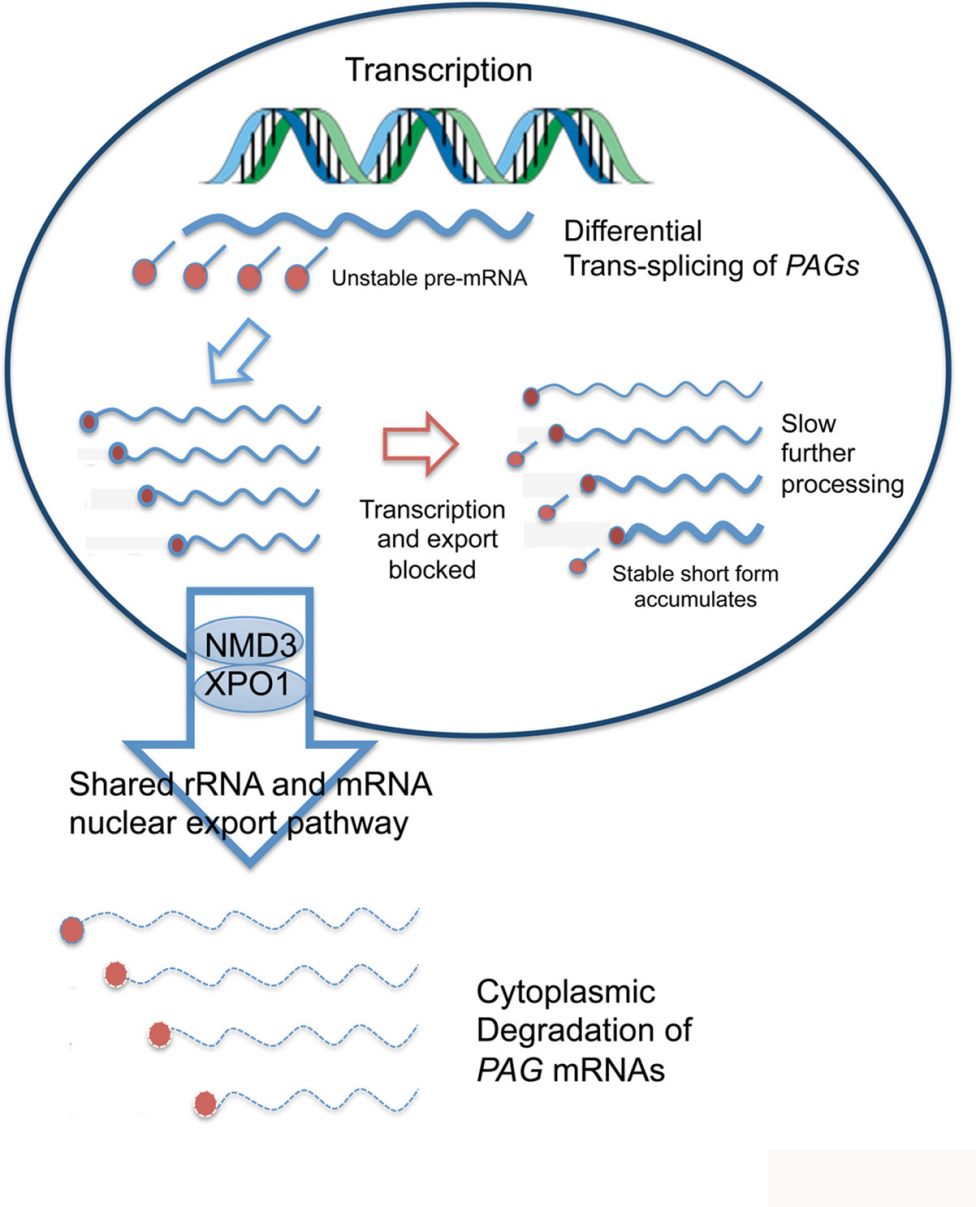
Model for the export and degradation pathway for *PAG* transcripts. The explanation of the model is provided in the manuscript text.

In Figure 9 we present a model that incorporates our findings based on the pathway for *PAG* transcript maturation and degradation. In this model, nascent *PAG* transcripts would be rapidly processed to a range of mRNAs of different lengths, which are then exported via an NMD3/XPO1-dependent process to the cytoplasm, where they are rapidly degraded, generating their relatively low abundance. With NMD3, XPO1 or MEX67 depletion, the newly synthesized and processed *PAG* transcripts would accumulate in the nucleus. When transcription is inhibited with Actinomycin D the accumulated nuclear transcripts could undergo further cycles of slower processing or differential nuclear decay, generating a short transcript form stable in the nucleus, with longer forms no longer being generated *de novo*. In response to inhibition of splicing by Sinefungin, *PAG* transcripts could be degraded in the nucleus by the nuclear exosome (59). Indeed, blocking *trans-*splicing may specifically activate the degradation of incompletely processed transcripts in the nucleus, thus preventing the export of deleterious aberrant mRNAs and explaining the reversal of *PAG* mRNA accumulation in NMD3-depleted Sinefungin-treated cells. This NMD3-dependent export pathway could preferentially target a subset of mRNAs, particularly the *PAG*s, explaining the apparent specificity of the accumulation of this transcript family. However our results favour a bulk export pathway, with *PAG* mRNAs preferentially accumulating due to their high turn over necessitated by their transcription as part of the pol I driven *procyclin* gene locus. This general mRNA export mechanism is supported by three lines of evidence. Firstly, *Tb*NMD3 depletion results in cells with an accumulation of polyA+ mRNA in the nucleus as detected by *in situ* hybridization. Secondly, qRT-PCR of nuclear and cytoplasmic mRNAs demonstrates that several transcripts become enriched in the nuclear fraction when *Tb*NMD3 is depleted by RNAi. Finally, depletion of the bulk mRNA export factor MEX67 recapitulates the increase of *PAG* transcripts observed with *Tb*NMD3 depletion (54). Combined these observations support a role for *Tb*NMD3 in the export of RNAs from the nucleus in addition to their role in 60S rRNA export. We do not yet know if this entails formation of a shared, possibly transient, complex involving *Tb*NMD 3/XPO1 and *Tb*Mex67 or the passing of rRNA and mRNAs between distinct complexes which can interact or interfere functionally. Nonetheless, interaction between the pathway for rRNA and mRNA processing and transport matches a shared role of the recently identified nucleolar protein NRG1 in rRNA processing and *GPEET* expression control (60) and is consistent with LMB treatment of *T. cruzi* cells that generates nuclear mRNA accumulation, albeit with specificity for some mRNAs (54).

Our experiments suggest that trypanosomes exhibit a shared platform for the export of rRNA and mRNAs, analogous to the capacity for the bulk mRNA export MEX67-Mtr2 complex to contribute to the export of ribosomal rRNA subunits (61). Indeed MEX67 can act as a suppressor of NMD3 mutants in yeast, underscoring the potential for functional overlap between rRNA and mRNA export in eukaryotic cells. This overlap may be particularly relevant for abundant RNAs where a quantitative export is necessary for their function or targeted elimination in the cytoplasm, or be necessary in trypanosomes where bioinformatics analysis suggests the presence of a reduced set of nuclear export proteins. Moreover, such nuclear export pathways could be a control step generating the differential abundance of co-transcribed transcripts. Whether this also has a regulatory function whereby the export of individual transcripts can be tuned depending on the physiological demands on the parasite, or developmental cues, represents an exciting area of future study.

## ACCESSION NUMBER

RNA-Seq data in this manuscript have been submitted to the GEO database with the accession number GSE67246.

## SUPPLEMENTARY DATA

Supplementary Data are available at NAR Online.

SUPPLEMENTARY DATA
