# Supplementary material for: NMD3 regulates both mRNA and rRNA nuclear export in African trypanosomes via an XPOI-linked pathway
Source: Nucleic Acids Res. 2015 Apr 14;43(9):4491–504. doi: 10.1093/nar/gkv330 (PMC4482084; doi:10.1093/nar/gkv330)
Supplement: SUPPLEMENTARY DATA [file supp_43_9_4491__index.html]

NMD3 regulates both mRNA and rRNA nuclear export in African trypanosomes via an XPOI-linked pathway — NMD3 regulates both mRNA and rRNA nuclear export in African trypanosomes via an XPOI-linked pathway — SUPPLEMENTARY DATA 

# NMD3 regulates both mRNA and rRNA nuclear export in African trypanosomes via an XPOI-linked pathway

## SUPPLEMENTARY DATA

**Files in this Data Supplement:**

- SUPPLEMENTARY DATA
- SUPPLEMENTARY DATA
