# Supplementary material for: NMD3 regulates both mRNA and rRNA nuclear export in African trypanosomes via an XPOI-linked pathway
Source: Nucleic Acids Res. 2015 Apr 14;43(9):4491–504. doi: 10.1093/nar/gkv330 (PMC4482084; doi:10.1093/nar/gkv330)
Supplement: SUPPLEMENTARY DATA [file supp_gkv330_nar-00639-a-2015-File011.docx]

**Supplementary Data Legends**

**Figure S1**

Growth profiles of procyclic and bloodstream form *T. brucei* induced to knock down the expression of *Tb*NMD3 by RNAi. The data from three independent growth assays is shown ±S.D.

**Figure S2**

1. Genomic organisation of the *PAG* genes in *Trypanosoma brucei*. Primer binding sites for transcription analysis in Figure S4 are also indicated.
2. Schematic diagram of the RNAs derived from *PAG1* through use of alternative 5’ splice sites in the *PAG1* 5’UTR. The map is derived from SL trapping and RNA-seq data available at TritrypDB and from ([20](#_ENREF_20))

**Figure S3**

Northern blot of *PAG1* RNAs after the induction of NMD3 RNAi

RNAs were extracted every 24h after NMD3 RNAi was induced by the addition of tetracycline (1µg/ml). The upper panel shows RNA hybridised with a probe detecting *PAG1*, the lower panel shows the equivalent ethidium bromide stained gel. The *PAG1* transcripts were clearly elevated after 48h, this increasing subsequently. The ethidium bromide stained gel reveals the presence of the rRNA precursor in the induced samples.

**Figure S4**

Histone occupancy of various gene loci in cells induced, or not, to deplete *Tb*NMD3 by RNAi.

1. For a number of genes, nucleosome occupancy in the relevant regions was monitored by histone H3 chromatin immunoprecipitation and quantitative PCR to assess histone association at each locus. Although there was decreased histone occupancy for many loci upon *Tb*NMD3 depletion this was not specific to the *PAG* locus.
2. Relative abundance of histone H3 in cells where *Tb*NMD3 is depleted or not by RNAi. No change in the overall abundance of histone H3 was detected.

**Figure S5**

Northern blots of RNAs derived from cells where NMD3 RNAi is induced, or not. In each set of samples the cells were incubated with Actinomycin D, Cycloheximide or Sinefungin at time 0h. The blots were hybridised with a number of different probes to detect *PAG1, PAG5, EP, GPEET, PPRI* and *zfp3*.

**Figure S6**

Quantitation of the abundance of *PAG1* and *EP* *procyclin* after treatment with Actinomycin D, Cycloheximide or Sinefungin. In each case the relative abundance is expressed relative to the abundance of the mRNA from the same cells prior to the addition of drug. For the *PAG1* transcript, only the 1.8kb transcript isoform was quantitated.

**Figure S7**

Leptomycin B treatment of procyclic forms generates increased *PAG* transcript abundance and recapitulates *Tb*NMD3 depletion.

1. Relative transcript abundance for several genes quantitated from northern blots under conditions of 1µg/ml leptomycin B treatment, or not. *PAG* transcripts exhibit strong upregulation.
2. Northern blots of the relative abundance of different gene transcripts from cells treated, or not, with 1µg/ml leptomycin B. *PAG* transcripts show up-regulation and the size of *TAO* is shifted slightly, reflecting the situation seen with *Tb*NMD3 depletion.

**Figure S8**

Induced and uninduced cell images are shown of cells where a pre-incubation with unlabelled Oligo(dT) was included (‘Blocked’) or not. The Oligo(dT) signal is shown in red, DAPI staining of the nuclear and kinetoplast DNA is also shown. Around 20% of cells in the induced sample exhibited nuclear accumulation of polyA+ RNA at 60-62h post induction of RNAi.

|  | 5’ primer | 3’ primer |
| --- | --- | --- |
| *PAG*1/2 5’ | GTTTCCAGGCTACAAACCGT | CCCTACCCGACACTTTCCTA |
| *PAG*5 5’ | CCCACAATCCTTCAACTCTTC | CACTTGGGAGGAAAGCAAAT |
| *PAG*2/2* 5’ | TTCCGCCAAATATCTTCCTC | GTTGTGTGGAGGGTTGGG |
| *PAG*4 | GGAAAGGAATTTGCAACCAT | GCCTTTATTACGGGAATCCA |
| GU2 | CGCAGCTGCACGTAGTTAAT | GGAGTGTTGCTTCCCACATA |
| NMD1probe | GAGAGATTGAAAGAACTTCTGGACTCG | CATTCCCGGTTGCATTGTAG |
| NMD2probe | GCGATGAGCAGCGCATC | GAATACACTCCCAAGTGCAACGC |
| NMD3probe | CCAAAGTTACTACCTGAAGC | GCGTTCAGCACAAGAAGGATCC |
| *PAG*1probe | AGAGGTTCTAATACACGATGTAACTTGGGAG | CAAAAATAACAACGCGAGGGTCCTCCTG |
| *PAG*2probe | CAGAGCAAACCTGGAGGAAGCAATTAG | GTCGTCCTTTCCCCAATTTCCGCC |
| *PAG*4probe | ATGACAACCATGGGGAAGACAATAAAAGGG | CCCCGTGAAGTTTTGGCGATCTTTGACACA |
| *PAG*5probe | ATGCGAAGAACAGTGGCATTTTTAGTAGTG | GGGTAGCACTTCTTCAGGGAAATTCACAC |
| TAO1probe | TTTCTTGAAACTGTTGCCGGT | CTGTTTTGCAGGCGTTTTTCG |
| PPR1probe | GTTTGGAGCCGGATGATTTAG | GAATATCGTCACTTAAGCCCT |
| Xpo1probe | GCTGGTTGGATGGTGAAGGGAGAG | GCCACAAAGTCACATTCTTCCGTATTGTC |

**Table S1**

Riboprobes for *zfp3*, *EP*, *GPEET, SmF, SmB* are described in references (39, 47). Probes for the *aldolase*, *ingi* and *Tb*11.47.0019 were provided by Drs. Katelyn Fenn and Stephanie Monk, University of Edinburgh.

**Supplementary Data 1**

RNA-Seq profile of procyclic form parasites induced or not to deplete TbNMD3 over 72h, Reads were mapped against the February 2015 genome version available at http://tritrypdb.org/tritrypdb/.


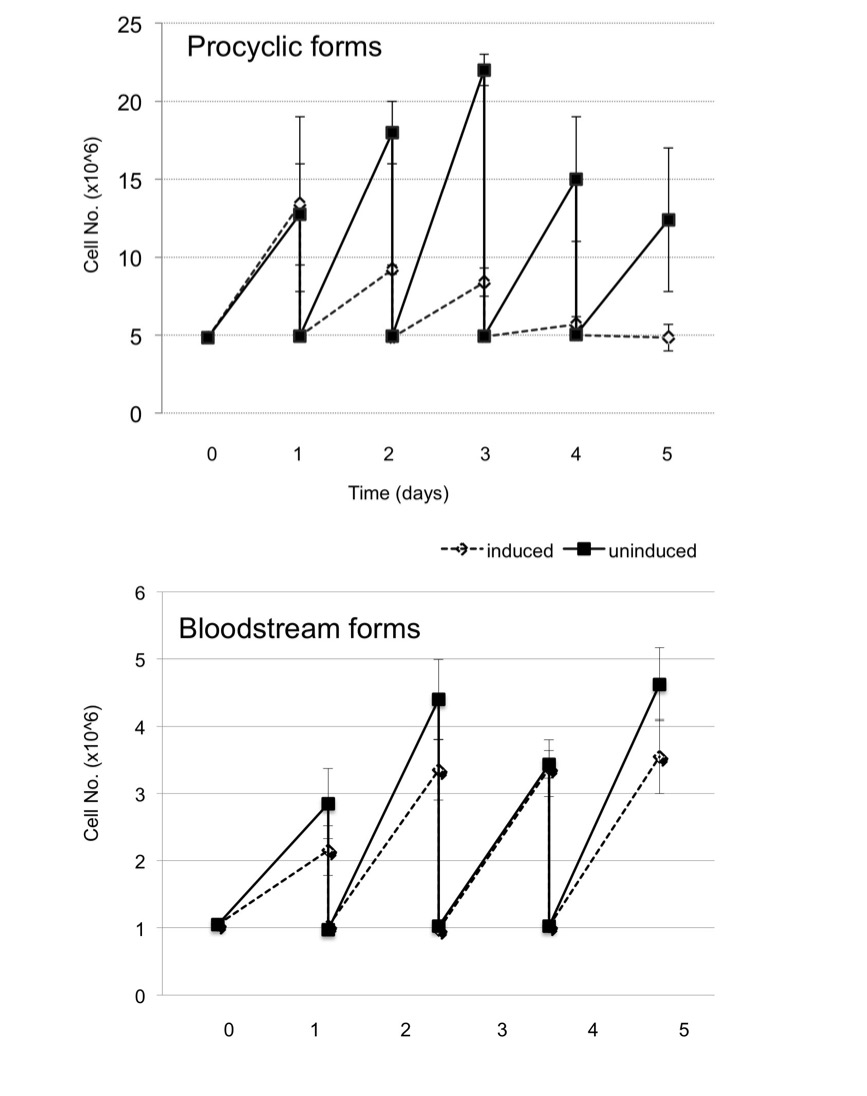


**Figure S1**


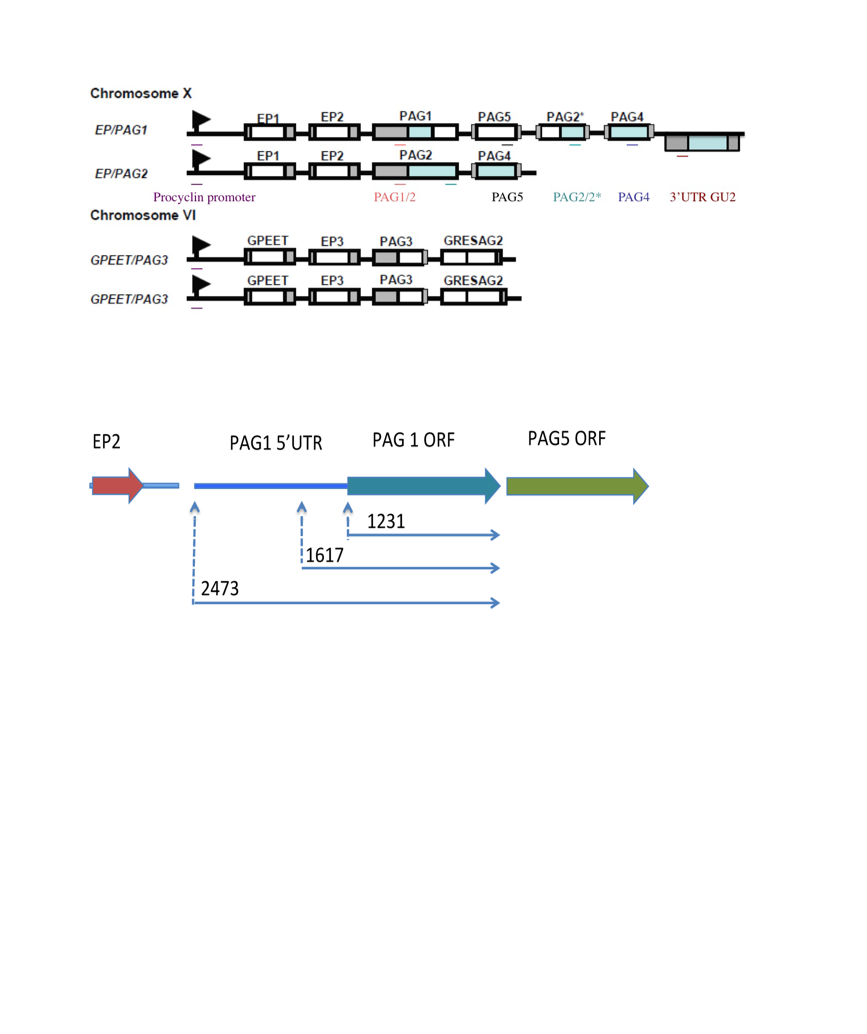


**Figure S2**


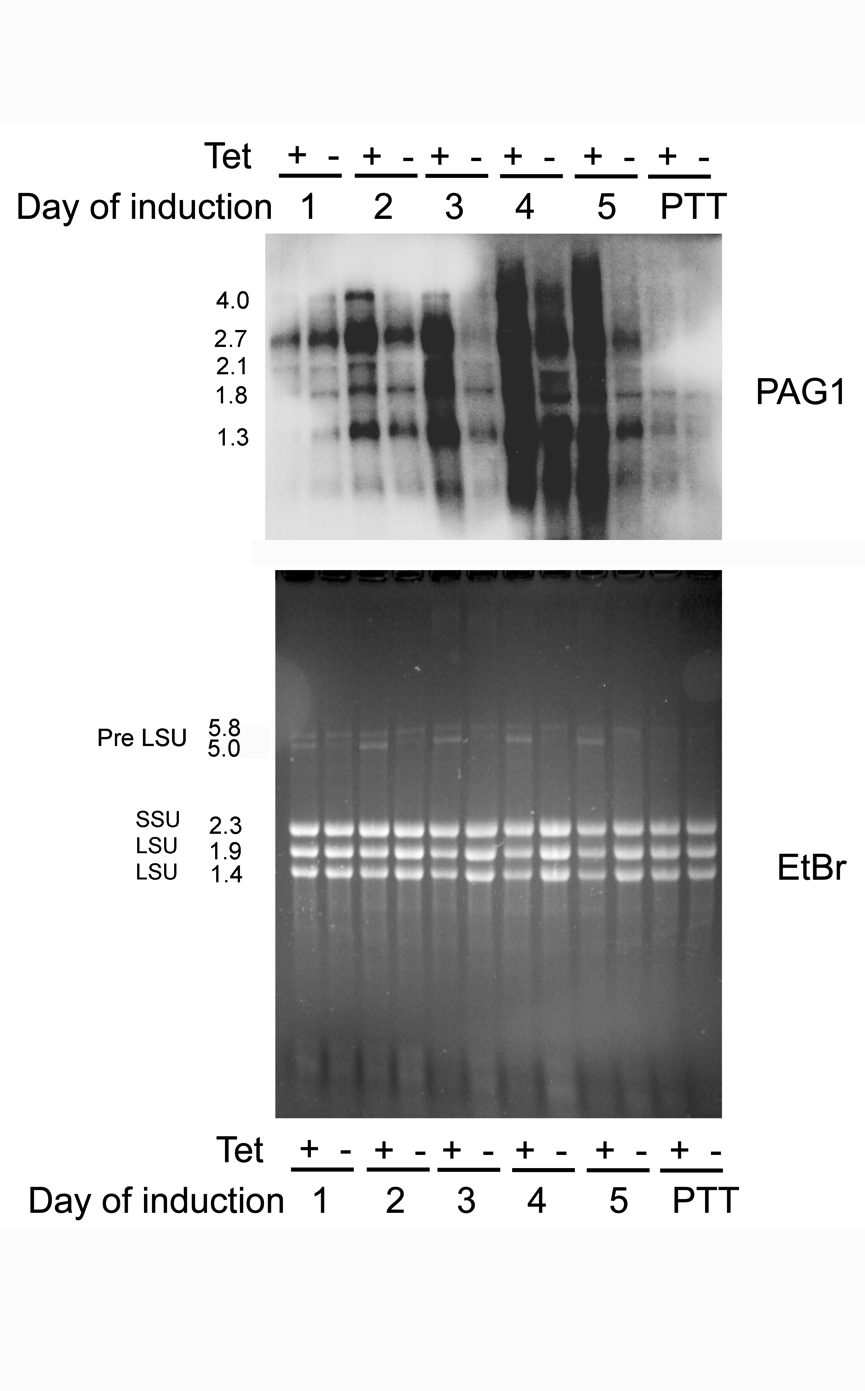


**Figure S3**


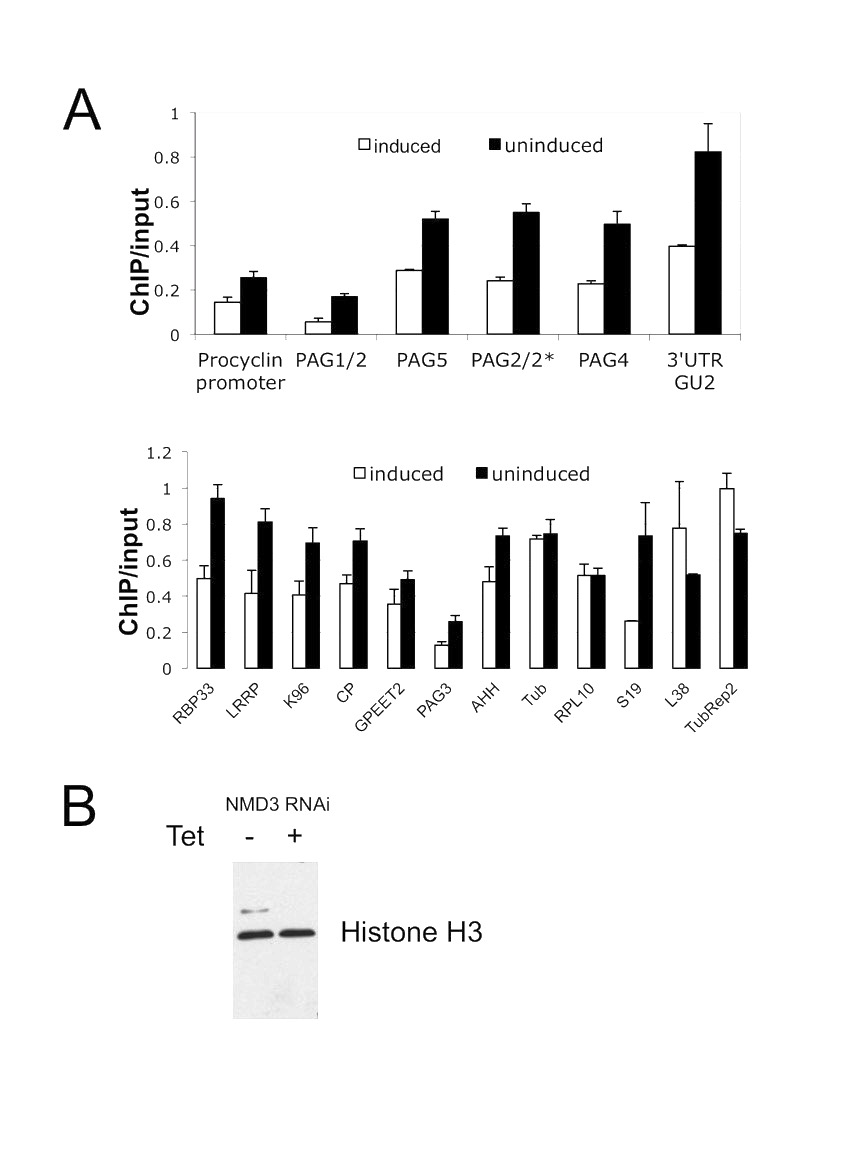


**Figure S4**


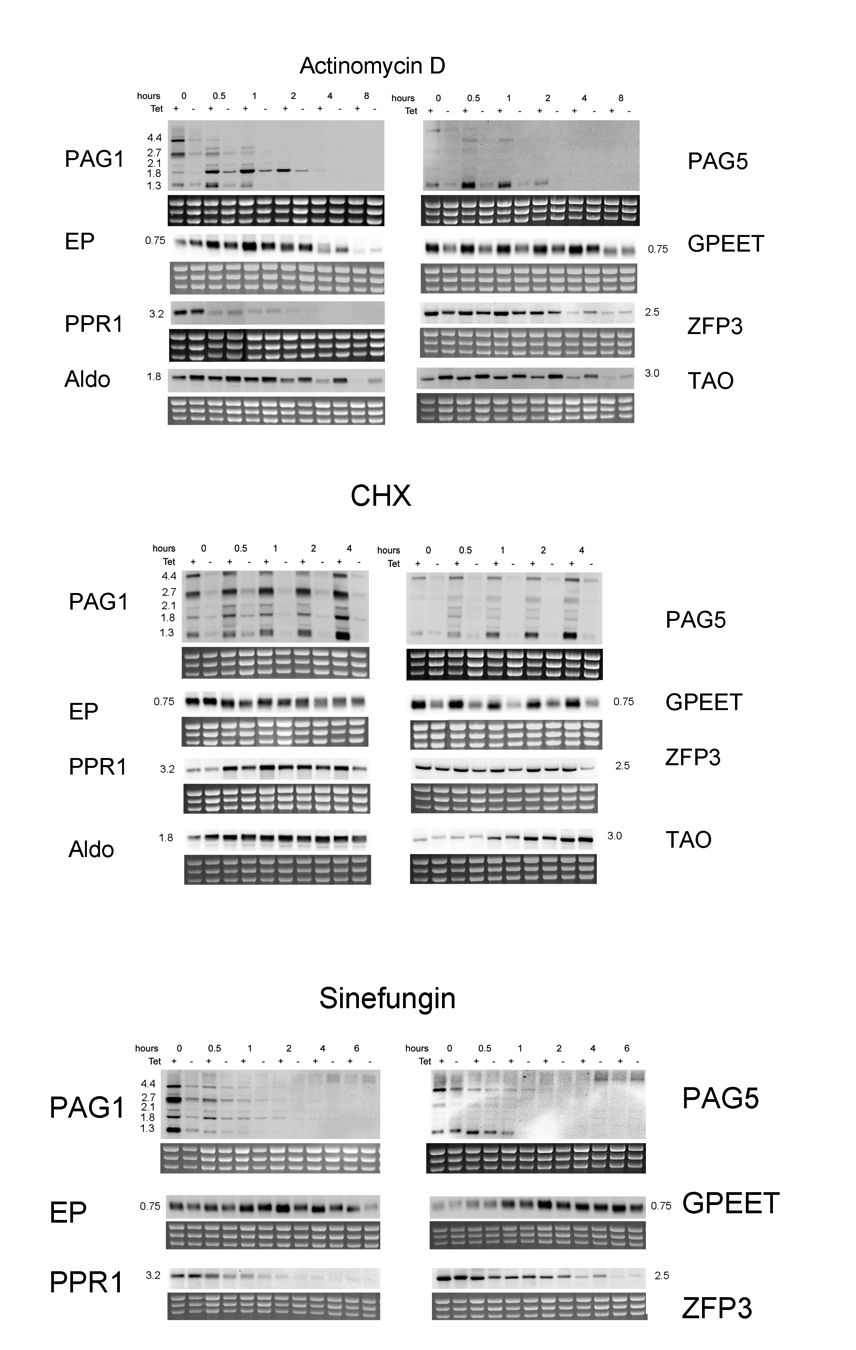


**Figure S5**


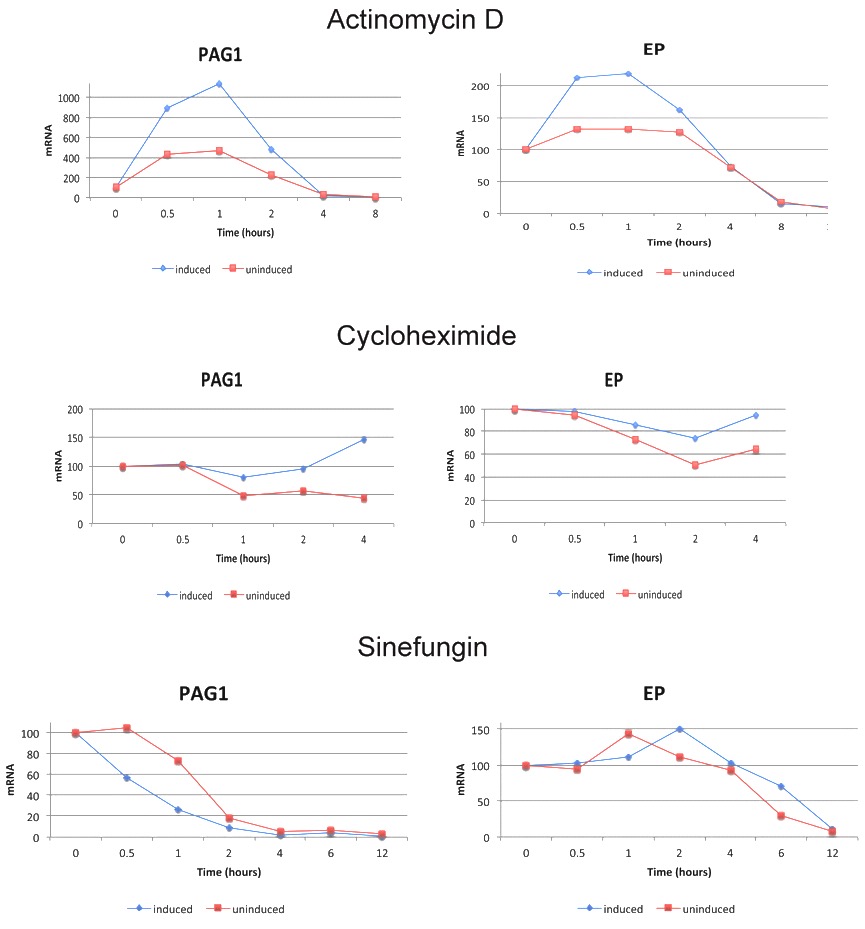


**Figure S6**


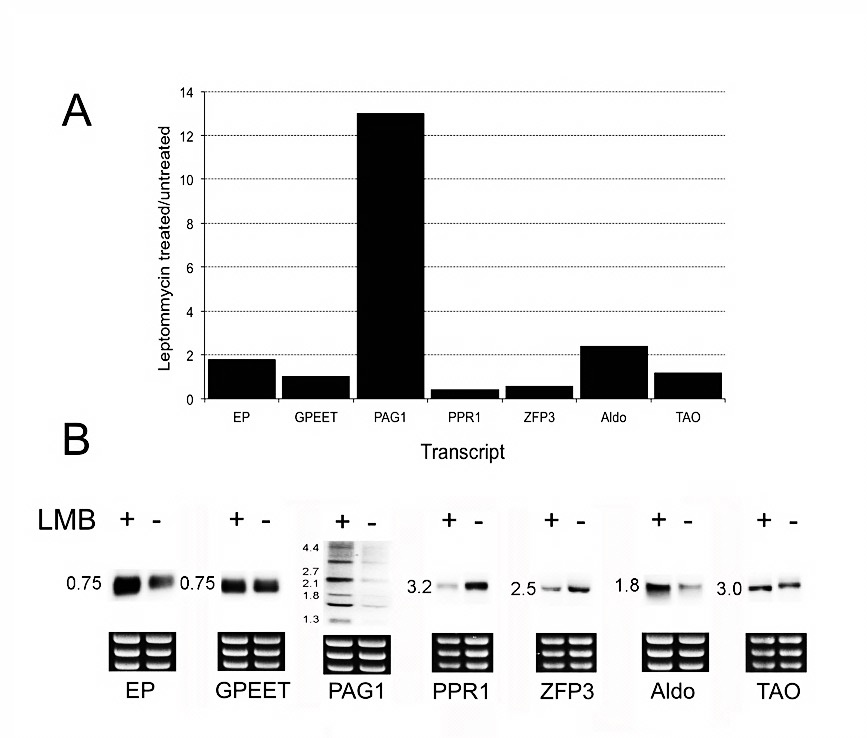


**Figure S7**


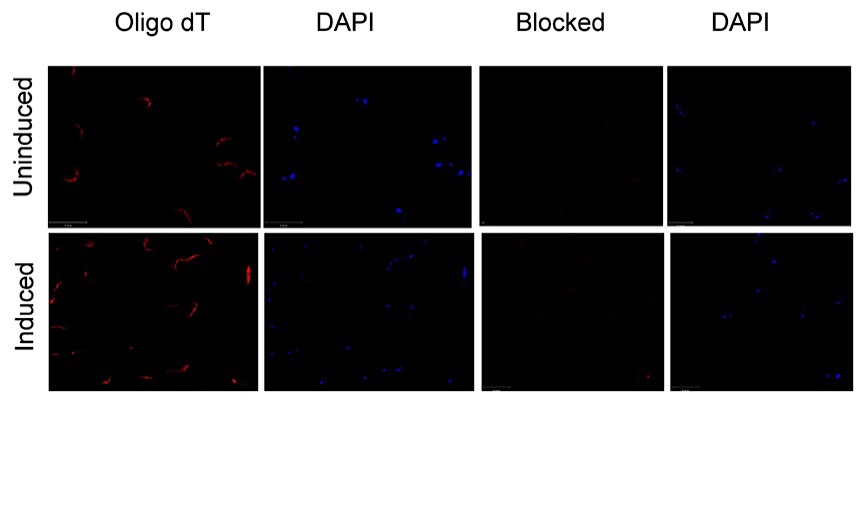


**Figure S8**
